# Cross-Hemispheric Genetic Diversity and Spatial Genetic Structure of *Callinectes sapidus* Reovirus 1 (CsRV1)

**DOI:** 10.3390/v15020563

**Published:** 2023-02-18

**Authors:** Mingli Zhao, Louis V. Plough, Donald C. Behringer, Jamie Bojko, Andrew S. Kough, Nathaniel W. Alper, Lan Xu, Eric J. Schott

**Affiliations:** 1 Institute of Marine and Environmental Technology, University of Maryland Baltimore County, Baltimore, MD 21202, USA; 2 Department of Pathobiology and Population Sciences, Royal Veterinary College, London AL9 7TA, UK; 3 Horn Point Laboratory, University of Maryland Center for Environmental Science, Cambridge, MD 21613, USA; 4 Fisheries and Aquatic Sciences, University of Florida, Gainesville, FL 32653, USA; 5 Emerging Pathogens Institute, University of Florida, Gainesville, FL 32608, USA; 6 School of Health and Life Sciences, Teesside University, Middlesbrough TS1 3BA, UK; 7 John G. Shedd Aquarium, Haerther Center for Conservation Research, Chicago, IL 60605, USA; 8 Baltimore Polytechnic Institute, Columbia University, New York, NY 20027, USA; 9 Department of Marine Biotechnology and Institute of Marine and Environmental Technology, University of Maryland, Baltimore County, Baltimore, MD 21202, USA; 10 Institute of Marine and Environmental Technology, University of Maryland Center for Environmental Science, Baltimore, MD 21202, USA

**Keywords:** RNA virus, phylogenetic, geography, host life history, climate, anthropogenic movement

## Abstract

The movement of viruses in aquatic systems is rarely studied over large geographic scales. Oceanic currents, host migration, latitude-based variation in climate, and resulting changes in host life history are all potential drivers of virus connectivity, adaptation, and genetic structure. To expand our understanding of the genetic diversity of Callinectes sapidus reovirus 1 (CsRV1) across a broad spatial and host life history range of its blue crab host (*Callinectes sapidus*), we obtained 22 complete and 96 partial genomic sequences for CsRV1 strains from the US Atlantic coast, Gulf of Mexico, Caribbean Sea, and the Atlantic coast of South America. Phylogenetic analyses of CsRV1 genomes revealed that virus genotypes were divided into four major genogroups consistent with their host geographic origins. However, some CsRV1 sequences from the US mid-Atlantic shared high genetic similarity with the Gulf of Mexico genotypes, suggesting potential human-mediated movement of CsRV1 between the US mid-Atlantic and Gulf coasts. This study advances our understanding of how climate, coastal geography, host life history, and human activity drive patterns of genetic structure and diversity of viruses in marine animals and contributes to the capacity to infer broadscale host population connectivity in marine ecosystems from virus population genetic data.

## 1. Introduction

### 1.1. Marine Disease Transmission

Epizootics appear to be increasing in many marine ecosystems, significantly impacting marine species and communities and prompting new research into the mechanisms behind how diseases are spread and influenced by host life history strategies [1,2]. Disease transmission, epizootics, and pathogen/host distributions have been extensively studied in terrestrial systems. However, our understanding of viral pathogen dispersal in the ocean remains limited despite well-documented epizootics over the past several decades. In coral reef ecosystems for example, epizootics have led to the die-off of the keystone reef herbivore *Diadema antillarum* [3] and have impacted corals indirectly [4,5]. Despite the apparent importance of disease in shaping marine ecosystems, there has been surprisingly little research on the population connectivity, genetic structure, and evolutionary dynamics of marine pathogens [6]. There are many studies using host population genetic structure to infer pathogen spread and dynamics in terrestrial systems (e.g., [7,8,9]). The genetic structure of pathogens is expected to parallel that of their hosts, since pathogen dispersal is often assumed to be driven by host dispersal, especially for host-specific and directly transmitted pathogens [9,10,11,12]. Conversely, the genetic structure and genetic differentiation of pathogens should reflect the movement patterns of their hosts, and thus could be a valuable proxy for host movement and population connectivity [8].

Barriers to dispersal and environmental conditions are important factors determining the biogeography of marine viruses and the co-regulation of marine viral community composition [13]. The ocean has fewer barriers to host and pathogen dispersal than terrestrial environments, which contributes to the wide and sometimes rapid spread of pathogens in marine ecosystems [14,15]. Marine organisms, including pathogens, can remain suspended and disperse over extended spatial scales due to the high density and viscosity of seawater, contributing to their characteristic long-distance dispersal (LDD) [16,17]. Marine invertebrates with a planktonic life stage have been historically described as having “open” populations because of their high LDD potentials [18,19,20,21], but recent research on larval transport and genetic patchiness over relatively small geographic scales has shifted consensus to a continuum of dispersal including LDD and local retention [22,23]. Host dispersal can also shape the genetic structure of pathogen populations by increasing the genetic uniformity between populations and thus decreasing global genetic diversity [24]. Similarly, human-mediated transport of hosts or pathogens to new areas alters the connectivity between their populations, potentially altering the magnitude of gene flow and the evolution of populations over time. The anthropogenic acceleration of marine pathogens is well known, and is documented in the intentional movement of marine animals between aquaculture facilities [25]. Examples include ostreid herpesvirus-1 microvariant (OsHV-1 µ-var) in Pacific oyster *Crassostrea gigas* [26,27] and Piscine orthoreovirus (PRV) in Atlantic salmon (*Salmo salar* L.) [28]. In addition to dispersal, a more complete understanding of marine pathogen biogeography should consider local or regional factors such as seasonal variation in host life history [29]. Variation of host life history can put selective pressure on pathogens [30], which in turn further drives host evolution, and shapes epidemiology, biogeography, and host/pathogen genetic structure [31,32,33].

### 1.2. Blue Crab–CsRV1 Pathosystem

The blue crab, *Callinectes sapidus*, is a valuable fishery species of the family Portunidae, along the western Atlantic coast from the US mid-Atlantic to Southern Brazil [34,35]. To match market demand with seasonal cycles of commercial crab harvests, intercoastal transport of blue crab is a common practice. *C. sapidus* is a highly adaptive and successful species with a wide climate and salinity tolerance. Its distribution encompasses a wide range of geographic scales, including its native habitats along the western Atlantic coast from Nova Scotia (Canada) through the Gulf of Mexico and the Caribbean Sea, to northern Argentina, and an extensive invasive range in Europe and Asia [36]. Blue crabs in the highest latitudes of temperate regions have life histories with prolonged winter dormancy, which becomes progressively shorter with decreasing latitude until the subtropics and tropics, where crabs are active year-round [37,38,39]. Blue crabs have a high potential for long-distance dispersal (LDD) [40,41]. Post-mating, female crabs can migrate tens to hundreds of km along the shore [42] or offshore [43] to reach spawning (larval release) sites. Planktonic larvae may travel many hundreds of km in coastal waters driven by large-scale ocean currents. Consequently, very low levels of genetic differentiation and high gene flow have been detected for the blue crab populations within the US (e.g., [44,45,46]). High-resolution genome-wide SNP data provided slightly greater resolution of population structure within the US and revealed large genetic separation between the Brazilian and the North American populations, suggesting limited gene flow between the two continents [47]. Based on the cytochrome c oxidase I (COI) mitochondrial sequence, significant genetic differentiation was also detected between blue crab populations in the US and Venezuela [48].

Callinectes sapidus reovirus 1 (CsRV1) is a pathogenic virus with a segmented dsRNA genome, originally identified in blue crabs captured from the Chesapeake Bay in the 1970s [49,50]. Segmented RNA viruses have high mutation rates, short generation time, and undergo segment recombination and reassortment, making them good models for studying evolution and population genetics [51]. CsRV1 has been shown to infect blue crabs along much of the crab’s western Atlantic, Gulf, and Caribbean range [2]. The prevalence of CsRV1 is greatest in higher latitude temperate zones and far lower in the subtropics and tropics, suggesting links between host life history and infection [2]. In temperate regions, winter-dormant crabs have lower CsRV1 prevalence than summer crabs suggesting interactions between blue crab life history, climate, and viral infection dynamics [2]. While CsRV1 is mainly found in blue crab along the Atlantic coast, it may infect *Macropipus depurator* in the Mediterranean Sea, based on the >90% sequence identity between CsRV1 and P virus found in *M. depurator* [52,53]. The transmission mechanism of CsRV1 has yet to be determined, but preliminary studies suggest a lack of vertical transmission from infected females to larvae.

Despite the association of CsRV1 with blue crab mortality and the observed relationship between latitude and CsRV1 prevalence in blue crab [1,2], little is known about the spatial patterns of CsRV1 genetic diversity. A prior study revealed substantial sequence divergence in the CsRV1 RNA-dependent RNA polymerase (RdRp) gene between the United States and Brazil [54]. Here, we examined CsRV1 genomes across the wide range of the blue crab distribution to investigate the genetic structure, genotypic diversity, and evolutionary dynamics of the virus in the marine environment. We use phylogenetic and population genetic analyses to infer virus genetic connectivity and movement over small and broad geographic scales, and explore the possibility that virus diversity is driven by host movement and human-mediated transportation of blue crabs. We examine virus genetic diversity in the context of a latitudinal gradient in blue crab life history to examine the effects of climate and host life history on virus evolution. We also analyze genetic diversity and selection pressure on each genomic segment to compare the evolutionary dynamics of 12 segments of CsRV1. Results of this study advance knowledge of genetic diversity and genetic structure of marine viruses across large spatial scales, and raise questions about the effects of host life history and human-mediated activity on viral transmission and population genetic structures.

## 2. Materials and Methods

### 2.1. Sampling

**Figure 1:**
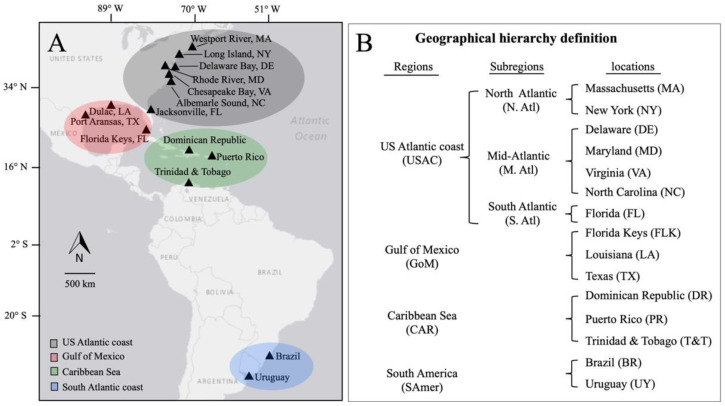
Map of sampling locations. (**A**) Shaded ellipses indicate larger geographic regions: US Atlantic coast (gray), Gulf of Mexico (red), Caribbean Sea (green), and South America (blue). (**B**) The geographic hierarchy definition of the sampling sites.

Blue crabs were collected from years 2015 to 2019 at 15 locations along the US Atlantic coast (USAC): Massachusetts (MA), New York (NY), Delaware Bay (DE), Maryland (MD), Virginia (VA), North Carolina (NC), and Florida (FL); the Gulf of Mexico (GoM): Texas (TX), Louisiana (LA), and Florida Keys (FLK); the Caribbean Sea (CAR): Dominican Republic (DR), Puerto Rico (PR), and Trinidad and Tobago (T&T); and South America (SAmer): Brazil (BR) and Uruguay (UY) (Figure 1; Table 1).

Leg samples were either placed on ice (locations within the US) or preserved in 95% ethanol or >120 proof white rum (locations outside the US) for shipping to the Institute of Marine and Environmental Technology (IMET) in Baltimore, MD, USA. At IMET, the samples were stored at −20 °C until analysis. RNA was extracted from ~50 mg of muscle and epidermis tissue from a walking leg and homogenized using a Savant MP^®^ FastPrep24 homogenizer with ceramic beads in 1.0 mL phenol-guanidine thiocyanate reagent (TRIzol, VWR scientific or equivalent) [55]. Extracted RNA was dissolved in 50 µL 1 mM EDTA and stored at −80 °C. CsRV1 positive specimens were then detected by quantitative reverse transcription PCR (RT-qPCR), as described in [2].

### 2.2. Sequencing

Sequence information of Segment 9 (seg9) was obtained by dideoxy sequencing (BigDye Terminator v3.1 Cycle Sequencing Kit, Thermo Fisher Scientific, Waltham, MA USA) of segment-specific amplicons. The primer pair used to amplify seg9 (960 nt amplicon) is listed in Supplementary Table S1, and PCR amplification was performed at 40 cycles of 95 °C for 5 s and 62 °C for 90 s, followed by 72 °C for 30 s. Sanger sequencing was performed in the BioAnalytical Services Laboratory at IMET.

Illumina sequencing was used to obtain whole genome sequences of CsRV1. Extracted total RNA was used for cDNA synthesis with barcoded random octamers (5′-GGCGGAGCTCTGCAGATATC-NNNNNNNN-3′), using M-MLV reverse transcriptase (Invitrogen, Waltham, MA, USA) according to the manufacturer’s instructions. The resulting cDNA was amplified by PCR using the primer (5′-GGCGGAGCTCTGCAGATATC-3′) [56]. PCR conditions were 40 cycles of 95 °C for 5 s and 60 °C for 30 s, followed by 72 °C for 30 s. RNA samples with low CsRV1 abundance were amplified through a multiplex PCR of synthesized cDNA, with specific primers designed according to the published whole genome of CsRV1 [54]. In total, 76 pairs of primers targeting nearly all of the CsRV1 genome were designed on Primal Scheme (http://primal.zibraproject.org (accessed on 6 September 2018)) (Supplementary Table S1) [57]. Each primer pair was designed to create a 400 bp PCR amplicon with a 75 bp overlap between neighboring amplicons. Multiplex PCR to amplify each segment was performed separately with the corresponding primer pools. PCR conditions were 40 cycles of 95 °C for 5 s and 58 °C for 30 s, followed by 72 °C for 30 s. Products of 250–500 bp were selected and obtained by agarose gel purification. DNA library preparation was performed using the NEBNext Ultra DNA Library Prep kit following the manufacturer’s recommendations (NEB, Ipswich, MA, USA). The library was sequenced in 2 × 250 paired-end configurations on the Illumina MiSeq platform at GENEWIZ (South Plainfield, NJ, USA) with a MiSeq Reagent kit v3 (Illumina, San Diego, CA, USA).

### 2.3. Sequence Editing and Alignment

Quality trimming and the removal of barcode sequences from raw reads were performed in CLC Genomics Workbench 9.5.2 (Qiagen, Hilden, Germany). CsRV1 sequences were identified by assembling reads against the published CsRV1 reference genome (MD-2008 #X45; Refseq. KU311708-KU311719) [54] in CLC Genomics Workbench. Assemblies were manually inspected and aligned with the GenBank genome sequences of the MD-2008 #X45 strain to identify sequencing gaps. Gaps were filled by dideoxy sequencing of amplicons created using the most adjacent flanking primer pairs from the multiplex set (Supplementary Table S1), and consensus sequences were exported for further analysis. Sequences of each segment were trimmed to obtain the same coding sequence length for all the samples. Each viral genomic segment was aligned using the L-INS-i algorithm implemented in MAFFT v.7.504 [58]. The concatenated genomes were generated with all the trimmed segments of CsRV1 in SequenceMatrix 1.7.8 [59]. The multiple sequence alignment of concatenated genomes from the collected 22 CsRV1 strains in this study and the genome of MD-2008 #X45 [54] were generated in MAFFT and presented in the NCBI alignment viewer. Amino acid sequences of all segment coding regions were aligned in MAFFT, concatenated in SequenceMatrix 1.7.8, and viewed in the NCBI alignment viewer.

### 2.4. Phylogenetic Analyses

Best-fit models for nucleotide substitutions were determined with Bayesian Information Criterion (BIC) using ModelFinder implemented in IQ-Tree 1.5.5 [60]. They were as follows: K2P + G4 for seg9 and TIM3 + F + I + G4 for the concatenated genomes. Maximum likelihood phylogenetic analyses were performed on each genomic segment based on models shown in Table S3 and the whole concatenated genomes using 1000 bootstrap replicates, with genome sequences of *Eriocheir sinensis* reovirus strain WX_2012 (SsRV WX_2012) [61] as the outgroup. Maximum likelihood phylogenetic trees based on amino acid sequences of CsRV1 were also constructed using IQ-TREE with 1000 bootstraps, and under the model of JTTDCMut + G4 for seg9 and JTTDCMut + F + I + G4 for the concatenated genome. Phylogenetic trees were then visualized and edited using FigTree v1.10.4 [62].

### 2.5. Spatial Patterns of Genetic Variability and Population Structure

Discriminant analysis of principal components (DAPC) was carried out in the adegenet 1.3–1 package for R studio (Jombart and Ahmed 2011), to infer the spatial genetic structure of CsRV1 and estimate membership probabilities of each viral sequence. For spatial genetic structure, the number of groups was pre-defined according to the geographic origins of CsRV1 samples: north Atlantic (N. Atl), mid-Atlantic (M. Atl), south Atlantic (S. Atl), GoM, CAR, and SAmer. The number of retained principal components (PCs) used for DAPC was selected by the a-score optimization method. The number of genetic clusters in the dataset (not grouping by sampling location) was also determined via K-means clustering in the ‘factoextra’ package [63], comparing multiple methods (Elbow, Silhouette, and Gap Statistics) to choose the most likely K. Values of K from 1 to 12 were tested.

### 2.6. Analyses of Nucleotide Diversity

Sequence alignments of both nucleotide and amino acid sequences were performed with MAFFT. Population diversity parameters, i.e., haplotype (H), haplotype diversity (Hd), nucleotide diversity (π), number of polymorphic sites (s), nucleotide differences (k), and average mutation rates (θ) were calculated using DnaSP v. 6.12.03 [64]. The ratio of non-synonymous (dN) to synonymous (dS) nucleotide substitutions (ω = dN/dS) was calculated with the HyPhy packages included in MEGA7 [65]. A dN/dS < 1 indicates the purifying selection, dN/dS = 1 indicates neutrality, and dN/dS > 1 indicates positive selection.

### 2.7. Neutrality Tests and Population Differentiation

Neutrality indices of CsRV1 populations were evaluated using the statistical tests Tajima’s D [66], Fu and Li’s F and D [67], and Fu’s Fs [68] implemented in DnaSP v.6.12.03 [64]. In these tests, positive values indicate a population contraction and/or balancing selection, while negative values denote an excess of low-frequency polymorphism and possible population expansion, which can result from a recent population bottleneck [69]. Harpending’s raggedness index (HRI) [70] and the sum of squared deviations (SSD) were also calculated using Arlequin v. 3.5.2.2 [71] to evaluate if the sequence data significantly diverged from the assumptions of a population expansion model. Small and non-significant values of HRI and SSD indicated a good fit between the observed and the expected values of the sudden expansion model and an expanding population [70,72].

Genetic subdivision of CsRV1 between sampling years, between regions, subregions, and locations, was assessed with the pairwise F*ST* statistics implemented in Arlequin v. 3.5.2.2. The relationship between genetic distance (F*ST*) and geographic distance was tested with a Mantel test with the package ade4 installed in R studio [73]. Geographic distance was measured as the Euclidean distance between locations in R studio. An analysis of molecular variance (AMOVA; 1000 permutations) was also run in Arlequin to analyze the population subdivision between collection time-periods, among regions of the US, CAR, and SAmer, between subregions within the US (N. Atl, M. Atl, S. Atl, and GoM), and between the locations within the SAmer (BR and UY).

## 3. Results

### 3.1. CsRV1 Genome Sequences

**Figure 2:**
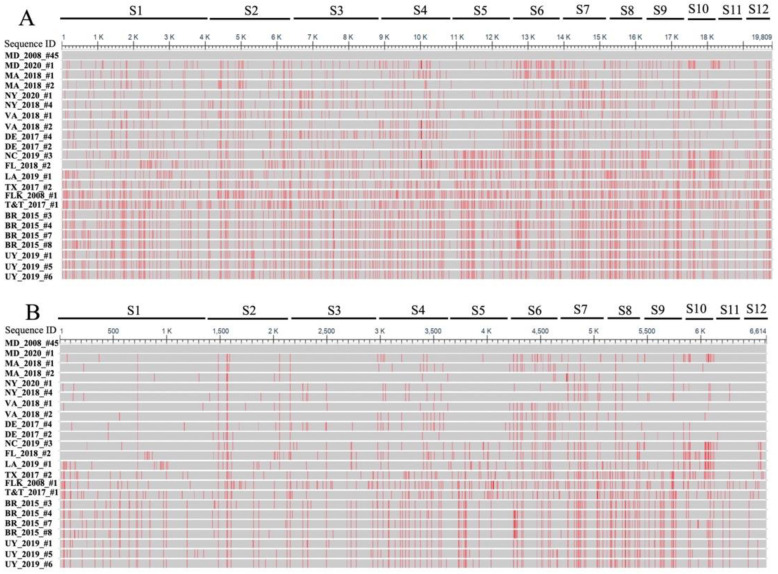
Alignment of concatenated CsRV1 whole genome nucleotide sequences (**A**) and translated amino acid sequences (**B**) visualized in NCBI alignment viewer. Red lines indicate differences from the MD 2008 X45 reference. S1–S12 indicate each of the 12 CsRV1 genome segments.

In total, 22 complete or near-complete genomes and 96 seg9 ORF sequences of CsRV1 were collected from 15 geographic locations along the US Atlantic coast (USAC), the Gulf of Mexico (GoM), the Caribbean (CAR), and South America (SAmer) between 2006 and 2021 (Table 1). The concatenated coding regions of all twelve segments (19,809 bp in total) for all 22 samples (hereafter referred to as ‘concatenated genome’) were compared to the published CsRV1 genome of MD-2008 #X45 [54]. The averaged sequence similarity between CsRV1 and the outgroup virus (SsRV WX_2012) was 64.4%. The nucleotide similarity across the 23 concatenated genomes of CsRV1 ranged from 97.3% to 99.7% (Figure 2A; Supplementary Figure S1A). In the USAC, virus strains sampled from different states had 98.4–99.5% nucleotide similarity within the region. In the GoM, CsRV1 genome sequences from TX and FLK shared 97.8% nucleotide similarity with one another. However, the genome of the LA strain (at the GoM) showed higher nucleotide similarity (>98.5%) with CsRV1 strains of the USAC rather than the GoM (<98.2%). The Trinidad and Tobago CsRV1 genome of the CAR had low nucleotide similarity with other geographic locations (97.3–97.9%). In SAmer, Brazil and Uruguay showed high nucleotide similarity within the region (99.3–99.7%), whereas they shared low sequence similarity with other locations (<98.6%) (Supplementary Figure S1A).

Comparison of concatenated genomes translated into coding regions (6614 AA) (Figure 2B) showed amino acid similarity across the 23 CsRV1 isolates from 96.8% (between FLK 2018#1 and LA 2019#1) to 99.7% (between UY strains). CsRV1 strains from MA, NY, DE, MD, and DE shared 98.9–99.7% identity. Lower amino acid sequence similarity (<97.2%) was revealed between the Gulf of Mexico and Caribbean regions, whereas higher sequence similarity was found within Brazil and Uruguay populations (>99.2%). Brazil and Uruguay had comparatively low similarity to the North American strains (97.2–98.1%) (Figure 2B; Supplementary Figure S1B).

Comparison of seg9 nucleotide and amino acid sequences among CsRV1 strains also revealed higher sequence identities within populations than between geographic populations of USAC, GoM, CAR, and SAmer. CsRV1 strains collected from GoM and CAR had overall lower within-population sequence similarities compared to viral strains within USAC and SAmer populations (Table 2).

### 3.2. CsRV1 Phylogenetics

**Figure 3:**
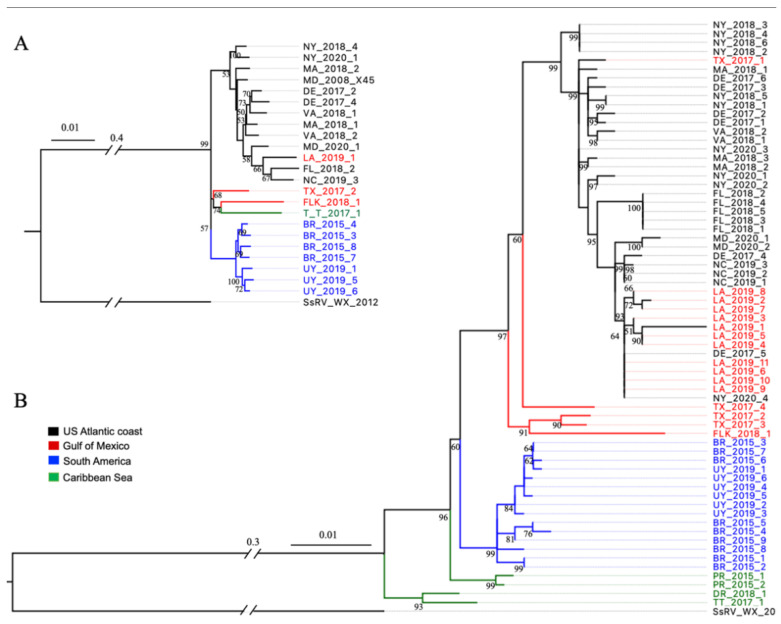
Maximum likelihood phylogenetic trees of CsRV1 collected from different geographic locations. (**A**) based on the nucleotide sequences of whole genomes (19,809 nt) and (**B**) seg9 (900 nt). Bootstrap values >50% are shown at each node of the tree, and branch lengths represent substitutions per site. SsRV WX_2012 was used as the outgroup.

The ML phylogenetic trees were reconstructed using the data for 23 concatenated genome sequences (Figure 3A) and 66 seg9 sequences (Figure 3B). The ML phylogenetic tree based on seg9 showed four branch clusters, with one of them not well supported (bootstrap 60). They were largely consistent with where the blue crab samples were collected, including group (I) USAC, (II) GoM, (III) SAmer, and (IV) CAR (Figure 3B). Group I encompassed CsRV1 genotypes from seven states along the US Atlantic coast, including MA, NY, DE, MD, VA, NC, and FL. Within Group I, genotypes of the six USAC states (MA, NY, DE, MD, VA, and NC) showed mixed distributions on the phylogenetic tree. However, FL CsRV1 strains formed a single clade separately from other USAC genotypes. Unexpectedly, a TX genotype and 11 genotypes collected from LA grouped with the USAC genotypes in Group I, rather than Group II, which included two TX genotypes and the FLK genotype from the GoM region. The isolates of BR and UY generated a single group (III), which was supported by high bootstrap values, and showed admixture between BR and UY strains on the ML tree. CsRV1 genotypes collected from CAR were divided into two clades, PR and T&T plus DR. These two clades together formed Group IV of the CAR genotypes.

The ML phylogenetic tree constructed from 23 concatenated genome sequences of CsRV1 showed a similar tree topology to Group I of USAC genotypes and Group III of SAmer genotypes (Figure 3A). Still, the genotype of the LA strain fell into Group I with the USAC genotypes instead of with the GoM genotypes. Whole genome sequences were obtained for only three CsRV1 strains from GoM and CAR due to the low CsRV1 prevalence in these areas [2], making it impossible to further detail the tree topology of CsRV1 concatenated genomes from these areas. However, it is inferred that these three genotypes form a distinct group from CsRV1 genotypes in other locations.

ML phylogenetic trees based on amino acid translations of seg9, and the concatenated genome translations, showed the same tree topology as the nucleotide sequences did, revealing four groups. Group I consisted of genotypes from the USAC and genotypes from TX and LA. Group II included genotypes collected from the GoM (TX and FLK). Group III included genotypes from SAmer (BR and UR), and Group IV included genotypes that originated from the CAR (Supplementary Figure S2A,B).

A ML phylogenetic tree was constructed for each genome segment. A comparison of the topology of the tree for each segment showed that positions of locations in Groups I and III were similar for seg3 and seg4, and seg7, seg8, and seg9, which was similar to the tree of the concatenated whole genome sequences (Supplementary Figure S3). For seg1, seg2, seg5, seg6, and seg10, genotypes of the USAC were too divergent to form a single group. For seg11 and seg12, nucleotide sequence divergence was not sufficient to generate a well-supported tree topology. Overall, more variations of tree topology were observed for GoM (TX and FLK) and CAR (T&T) genotypes among segments, indicating high genetic variation in these regions. However, there was insufficient data (low bootstrap support) to infer whether there was evidence for the reassortment of segments.

### 3.3. Genetic Diversity and Selection

Population genetic parameters were calculated for all 12 segments. Based on the seg9 sequences, the nucleotide diversity was higher in the GoM and CAR (π = 0.0135 and 0.0172, respectively) compared to the USAC and SAmer (π = 0.0078 and 0.0037, respectively) (Table 2). More synonymous than non-synonymous changes (ω < 1) were detected at all sampling locations (Table 2), suggesting purifying selection on the CsRV1 genome in all geographic populations. However, ω was larger for CsRV1 collected from the GoM and CAR than the USAC and SAmer (Table 2). Although the nucleotide diversity of all 12 segments was low (π < 0.021) for CsRV1, a higher nucleotide diversity can be observed for seg7, seg8, and seg10 compared to other segments (Table 3). The strength of negative selection was weaker on seg7, seg8, and seg10 (ω = 0.3~0.4) than on other segments, especially seg1, seg2, seg3, and seg11 (ω = 0.08~0.11). Average mutation rates (θ) were higher for seg7 and seg10 (0.031 and 0.037) compared to the other 10 segments (Table 3).

### 3.4. Neutrality Tests and Genetic Differentiation

Neutrality tests on genomic segments of CsRV1 were estimated using three statistical methods (Tajima’s D, Fu and Li’s D and F, Fu’s Fs) (Table 4). Test values for all 12 segments were negative, suggesting a purifying selection on CsRV1, and possible population expansion. Most values were non-significant, but significance was observed for seg1 through seg4, seg11, and seg12 for Fu and Li’s tests. In addition, both SSD and HRI values were low and non-significant for all genomic segments of CsRV1 (Table 4), consistent with the rapid demographic expansion of CsRV1 populations in the recent past.

To test the genetic differentiation of CsRV1 among geographic regions, F*ST* was calculated based on seg9 sequences. Pairwise comparisons between USAC and SAmer produced the largest estimates of genetic differentiation (F*ST* = 0.69), which indicates little to no gene flow between the two continents (Table 5). The F*ST* value between the GoM and USAC (=0.16) was much lower than between the GoM and CAR (=0.51) or the GoM and SAmer (=0.63), suggesting potential high CsRV1 gene flow between the GoM and USAC within the US, but very limited gene flow between the GoM and CAR or SAmer. CAR showed a similarly high genetic differentiation from both the USAC (F*ST* = 0.66) and the SAmer (F*ST* = 0.62).

Genetic differentiation was also investigated on a finer geographic scale between subregions within the US: N. Atl, M. Atl, S. Atl, and the GoM. Pairwise comparisons between the N. Atl and M. Atl showed lower F*ST* (0.23), indicating more gene flow between the two regions. A larger F*ST* was detected between S. Atl and other subregions, especially the N. Atl (F*ST* = 0.57), indicating a distinct genetic composition of S. Atl CsRV1 compared to other subregions in the US. Incongruously, the GoM showed the lowest genetic differentiation (F*ST* = 0.15) with M. Atl, but the highest genetic differentiation with S. Atl (F*ST* = 0.35).

**Figure 4:**
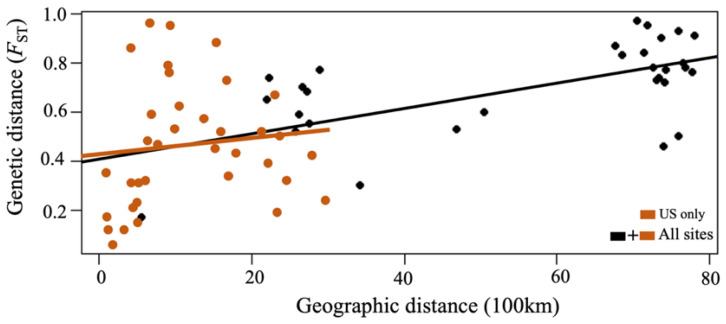
Isolation by distance. Genetic distance of AMOVA-based F*ST* versus geographic distance (geographical distance in ×100 km) is plotted for all pairwise comparisons among populations of the CsRV1 based on seg9 nucleotide sequences (843 nt). The black regression line is based on all the points, and the orange line is for only US sites. A correlation was detected with a Mantel test (*p* < 0.01 for all sampling sites; and *p* > 0.1 for only US sites).

Within the subregion of SAmer, BR and UY showed F*ST* estimates of 0.17, but the F*ST* estimates between BR/UY and locations of the USAC were higher than 0.72 (Supplementary Table S2. Unexpectedly, LA showed a very low F*ST* with MD (0.34), suggesting a high gene flow between the two locations. TX showed lower F*ST* estimates with the northern locations (e.g., MA and VA) within the subregions of N. Atl and M. Atl, but much higher F*ST* with FL and LA (Supplementary Table S2). Subsequently, the Mantel tests based on F*ST* estimates between locations showed a significantly positive association between the genetic distance (F*ST*) and the geographic distance (km) for all the sampling sites (*p* < 0.01), but not when considering the US sites only (*p* > 0.1) (Figure 4). When the geographic distance was restricted to the US, the Mantel test indicated no evidence of significant isolation by distance.

The AMOVA test on CsRV1 across the regions of the US (USAC & GoM), CAR, and SAmer revealed greater variance among populations than within the populations (Table 6). With AMOVA statistical values > 0.50 and *p* < 0.01, the F*ST* results based on seg1, seg8, and seg9 all allowed us to refute the null hypothesis of non-differentiation of CsRV1 between these geographic locations. AMOVA also suggested that there was no significant genetic differentiation of CsRV1 among populations within the US or within the SAmer populations. A higher percentage of variation was explained by the within-population components rather than among populations within each region.

### 3.5. Spatial Genetic Structure and Admixture Patterns

**Figure 5:**
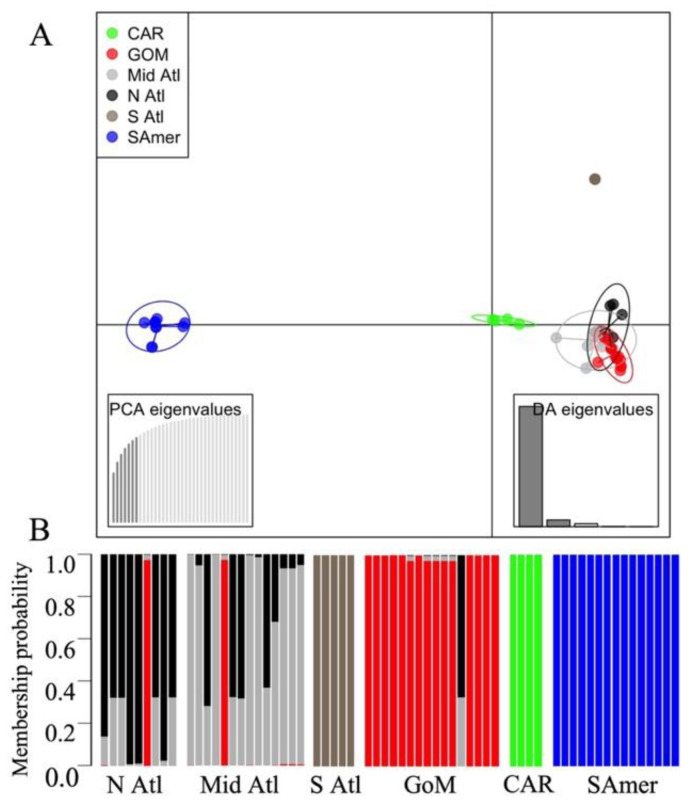
Discriminant analysis of principal component (DAPC) and membership probability based on seg9 nucleotide sequences of CsRV1. (**A**) DAPC scatter plot. Seven PCs and five discriminant eigenvalues were retained during analyses, to describe the relationship between the populations. Dots represent individuals and circles show clusters and colors indicate sampling origins (top right). Axes correspond to the first two discriminant functions. (**B**) The probability of membership of each sample in the six assigned groups. Bar colors represent the membership probabilities to various regions, the same as in the DAPC plot.

Seven principal components (78.7% of variance conserved) of PCA and five discriminant eigenvalues were retained for DAPC analyses with seg9 sequences based on the a-spline optimization results. K-means cluster analysis revealed that the most likely number of clusters (K) in the data was 4–5 based on a consensus of three metrics used to infer the most probable K (Elbow, Silhouette, and Gap Statistic Method). The scatter plot in Figure 5A shows the first two linear discriminants for all analyzed samples using the sample locations (*n* = 6) as the pre-defined clusters. All the CAR and SAmer genotypes were placed into distinct groups, and the SAmer showed the largest distance relative to other groups. Within the US, the S. Atl genotypes (exclusively collected from FL) grouped far away from the N. Atl and M. Atl populations. The N. Atl, M. Atl, and GoM populations appeared to overlap substantially with each other. Consistently, the membership probabilities of each genotype show two unique groups of the CAR and SAmer genotypes (Figure 5B). US N. Atl and M. Atl strains showed potentially high levels of admixture or equal probability of membership to either population, whereas the S. Atl samples were more distinct and had high membership probabilities to their “home” population. The GoM genotypes formed a single group, but some genotypes had apparent overlap with North and Mid-Atlantic genotypes and showed strongly deviating membership probabilities relative to their population of origin. For example, two genotypes of the M. Atl and N. Atl were assigned to GoM, and one individual from GoM was primarily assigned to N. Atl or M. Atl. Furthermore, potential admixture between CsRV1 from M. Atl and GoM was suggested for some genotypes (Figure 5B).

## 4. Discussion

Understanding the connectivity of marine crustacean populations and how their pathogens spread over large ocean distances remain fundamental questions in marine ecology. RNA viruses are particularly valuable models for the study of these processes due to their potential for rapid evolution and high genetic variability [74]. In this study, we found that CsRV1 sequences have pronounced differences over large regional scales, implying limited connectivity, but low sequence diversity, hence relatively high population genetic connectivity, over smaller, within-region geographic distances. We observed different within-region CsRV1 diversity in crabs from temperate versus subtropical/tropical locations. Some CsRV1 genome segments displayed notably higher degrees of genetic variation than others. Below we discuss the phylogenetic and population genetic observations of CsRV1 in the context of how geography, human-mediated transport of hosts, and host life history may impact observed patterns of CsRV1 genetic diversity and evolution. We also discuss the covariation of the population genetic structure of the virus and its host, based on prior literature on blue crab population genetics, and consider the potential for using population genetic analyses of marine pathogens to infer the movement and population connectivity of hosts with complex life cycles.

### 4.1. Genetic Differentiation between CsRV1 Populations over Large Geographic Scales

The phylogenetic lineages of CsRV1 were consistent with their host origins at the hemispheric scale of its distributions. CsRV1 genotypes can be divided into four genogroups: (I) US Atlantic coast (USAC), (II) Gulf of Mexico (GoM), (III) Caribbean Sea (CAR), and (IV) South America (SAmer). Genetic differentiation (F*ST*) among CsRV1 strains showed significant positive correlations with the geographic distance over the entire sampled range (7800 km). Population genetic analyses revealed high genetic differentiation (F*ST*) between the USAC and SAmer, suggesting very limited gene flow for CsRV1 between the two continents which is a trend consistent with that of the host populations (e.g., [46,47,48]). Analyses of the GoM and SAmer CsRV1 populations also revealed distinct patterns of genetic structure based on DAPC analyses and estimates of population genetic differentiation. Consistent with this, strong genetic differentiation between blue crab populations in the GoM and southern Brazil has been reported [46,47,75], which supports the congruence of patterns of population genetic structure between the pathogen and its host. Few genotypes of CsRV1 were collected from CAR due to the low CsRV1 prevalence in the Caribbean region [2]. However, seg9 genotypes from the CAR (PR, T&T, and DR) were grouped separately from other geographic regions. Distinct genetic structure was also observed for CAR populations in DAPC analyses, and the high estimated F*ST* values (and inferred low gene flow) confirmed the substantial genetic differentiation between CsRV1 from CAR and other geographic locations.

Prior reports of significant genetic differentiation of blue crab hosts between the Caribbean Sea and South America are not surprising due to the large geographic distance and biogeographic barriers between the two continents [47]. The Amazon River Plume acts as a soft barrier to blue crab larval transport and dispersal in the western tropical Atlantic, as the northwestward currents hinder the movement of larvae from the Caribbean to Brazil [76]. Coastal barriers to larval dispersal, such as river plumes, can affect population connectivity and gene flow, and may be common features in the ocean affecting a wide range of marine organisms with pelagic larval stages. For example, in the spiny lobster *Panulirus*, which has a long-lived larval stage, the Amazon–Orinoco plume differentiates the species into *P. meripurpuratus* sp. nov. of Brazilian waters from *P. argus* of North American waters and the Caribbean Sea [77,78].The Amazon River produces a surface plume of low salinity, which may act as a barrier to dispersal. However, the overall lack of freshwater input in tropical Northern Brazil results in large stretches of inhospitable habitat for blue crab, which may also contribute to the observed lack of connectivity over this region. [79,80]. The separation of blue crab populations by the Amazon River and the lack of blue crabs in tropical Brazil may well explain the distinct spatial genetic structure of CsRV1 between the Caribbean Sea and South America too. In the Caribbean, the largely unidirectional Caribbean currents (from east to west) and Antilles currents (from south to north) may significantly impact the genetic pattern of blue crab and CsRV1 genetic variation between the Caribbean and the Atlantic Ocean.

### 4.2. Limited Genetic Differentiation Found over Small Geographic Scales

Over smaller spatial scales, CsRV1 also showed lower genetic differentiation among sampling locations. There was no significant correlation between the genetic distance and geographic distance within the US (including the US Atlantic coast and GoM), suggesting high gene flow among CsRV1 populations located along a contiguous coastline. Genetic differentiation was also absent for CsRV1 between US north Atlantic and mid-Atlantic populations, and between Brazilian and Uruguayan populations. Ocean currents are the predominant factors enhancing larvae dispersal of blue crab, which consequently increases the population connectivity of crab hosts [81,82,83,84]. For example, the reported high level of gene flow among blue crab populations in southern Brazil was likely influenced by local currents, which facilitate the dispersal of larvae between locations [85]. Similarly, published analyses of blue crab genetics revealed very low genetic differentiation and high gene flow at fine geographic scales within the Gulf of Mexico, within southern Brazil, and along the US Atlantic coast [44,46,85,86].

Since vertical transmission has not been demonstrated for CsRV1, and the virus is unlikely to be carried and transported by blue crab larvae, the low genetic differentiation within these geographic regions suggests that the virus is carried by juvenile and adult blue crabs, which can travel tens of km along the near shore ([42]) and offshore [43]. Thus, post-larval crab migration has the potential to spread the virus within adjacent populations and waterbodies along the continuous coastlines of the US Atlantic coast and between Brazilian and Uruguayan crab populations. The scenario might be different in the discontinuous island habitats of the Caribbean Sea. Over a broader scale, continuous pelagic habitats are inhospitable to crabs except during their larval phase, which has yet to be shown to harbor the virus. Thus, areas with contiguous coastlines are expected to foster virus connectivity better than islands. The dispersal of crab larvae among localities in the Caribbean should be largely continuous and unidirectional under the prevailing Caribbean Current. However, persistent offshore gyres and counter-currents can prevent the dispersal of larvae and significantly increase the retention of larvae, as observed in the spiny lobster populations in the Caribbean [87,88]. To adequately compare the genetic structures of CsRV1 and its crab host in the discontinuous habitats of Caribbean islands would require the collection of more CsRV1 genotypes in the Caribbean Sea, where the virus prevalence is already very low [2].

### 4.3. Anthropogenic Transport of CsRV1?

The presence of closely related virus genotypes in widely separate geographic regions may indicate high connectivity between them, or could be the result of an incomplete lineage sorting after an ancestral split. The shared CsRV1 genotypes present in both USAC and GoM populations suggest that the GoM or USAC could serve as a potential CsRV1 dispersion point from one region to the other. Pathogen spread can be driven by natural processes of host movement or by human-mediated transport [89]. Long-distance dispersal of blue crab host genotypes, presumably by larvae, results in low genetic differentiation (high connectivity) between distant blue crab populations of the GoM or US northwest Atlantic [47,90]. Because there is no evidence of CsRV1 transmission in larvae, it is unlikely that larval transport has dispersed CsRV1 genotypes over this spatial scale. In addition, the significantly higher genetic differentiation between the GoM and S. Atl population (F*ST* = 0.35), compared to the GoM and M. Atl (F*ST* = 0.15), suggests that CsRV1 genotypes more likely moved directly between the GoM and M. Atl in a small number of individuals, rather than through the S.Atl. It is highly unlikely that CsRV1 genotypes moved from the GoM to the M. Atl region by coastal crab migration without leaving genetic evidence in the S. Atl. Similarly, it is difficult to explain these F*ST* differences as a result of an incomplete lineage sorting from a past split.

A plausible explanation for the similar CsRV1 genotypes is that they were received via the importation of infected blue crabs from other regions. The similarity of CsRV1 genotypes in the GoM and M. Atl is entirely consistent with the long-distance movement of CsRV1 mediated by interstate trucking of virus-infected crabs that are being seasonally traded between the M. Atl and GoM. Confidential communications with crab fishermen, soft crab producers, and seafood dealers in Maryland and Louisiana reveal that the scale of interstate transport is large yet un-measured. In both winter and summer months, blue crabs are shipped to the M. Atl regions because of the high consumer demand. Pre-molt blue crabs (a.k.a. peelers) also travel among states in the US as the ratio of peelers and soft-shell crab production capacity shifts between the regions. The stresses of long-distance transportation and the process of soft-shell crab production can concentrate virus-infected crabs. Moreover, it is common for soft crab producers and dealers to dispose of dead imported crabs in local estuaries. Therefore, human-mediated dispersal of CsRV1 during the transportation of blue crabs is a more parsimonious explanation for the high genetic similarity of CsRV1 between these two regions. Similar lessons have been learned from the global spread of OsHV-1 and OsHV-1 µ-var, which was linked to the human-mediated movement of Pacific oysters and insufficient biosecurity practices [27,91].

### 4.4. Genetic Diversity, Variable Environment and Host Life History

Host life history across a variable environmental range has the potential to affect pathogen genetic diversity and genetic structures [30]. Blue crabs in temperate regions seasonally migrate to deep water or burrow into estuary sediments to overwinter, while crabs in the subtropics and tropics have year-round activity [37,38,39,92]. A much higher genetic diversity of CsRV1 was revealed in crabs from the tropical and subtropical GoM and CAR compared to the temperate USAC and SAmer populations. Viruses are reliant on active host metabolism to replicate and spread, making host life history, population size, and distribution important determinants of the genetic diversity of viral populations. Therefore, the higher genetic diversity of CsRV1 in the tropics and subtropics could be associated with the year-round activity of blue crabs at these latitudes. High temperature could enhance the replication of the virus, and the year-round active host life history provides more opportunities for CsRV1 to replicate. Both factors could then lead to more mutations and the higher genetic diversity of CsRV1 in the tropics.

In addition, behaviors unique to blue crabs in temperate regions, such as seasonal migrations for winter dormancy and mating activity, may increase contact between crabs from distant locations and therefore decrease the genetic differences of CsRV1 genotypes among locations. Previous work revealed that CsRV1 prevalence was significantly correlated with temperature and seasons [2]. The lower CsRV1 prevalence but higher CsRV1 genetic diversity detected in GoM and CAR may indicate complex effects of temperature and life history on virus genetics and epizootiology. More study is needed to generate and test hypotheses about the relationships between CsRV1 transmission, prevalence, genetic variation, and virulence, with environmental conditions and host life history. It is likely that transmission rate, virulence, and disease progression of CsRV1 differs in blue crabs in temperate versus subtropical/tropical regions, driven in part by geographically distinct CsRV1 strains and genetic differences in the host.

Besides the impact of latitude and host biology, geographic factors also likely contribute to the evolutionary dynamics of the CsRV1. Studies revealed the restricted population connectivity and short-distance dispersal of coral (*Acropora palmata*) and sponge (*Ircinia campana*) larvae in the Caribbean, limited by geographic distances among habitats [93,94]. Similarly, blue crab habitats in the CAR areas are also likely to be isolated islands linked by larval transport, with fewer opportunities for juvenile or adult migration between islands. This would substantially reduce the movement of juvenile or adult blue crabs between populations, and thereby interrupt the population connectivity of CsRV1 (increased differentiation). In contrast, blue crab habitats along the USAC and SAmer are more contiguous, enabling greater interactions among populations and facilitating migration along the coast that would reduce genetic differentiation among locations.

### 4.5. Variable Evolution among CsRV1 Genomic Segments

All segments of the CsRV1 genome appear to be under strong purifying selection, to different degrees. Observations of purifying selection in genomic segments have been reported for other reoviruses, such as the bluetongue virus [95]. Tajima’s D and Fu and Li’s tests also indicated negative selection on all the CsRV1 segments. The non-significant pairwise mismatch distributions suggest that CsRV1 has probably undergone rapid population expansion. This expansion is consistent with poleward range expansion since the last glacial maximum [96]. Notably, the neutrality tests were only significant for Segments 1–4 and Segments 11–12, indicating a stronger power of negative selection on these segments. In contrast, neutrality tests were not significant, and relatively higher dN/dS values were observed for Segments 7, 8, and 10, suggesting that proteins translated from these segments were under periodic positive selection. Correspondingly, seg1 and seg2 encode proteins with highly conserved functions: RNA-dependent RNA polymerase and guanylyltransferase, respectively. Segment 8 putatively encodes the viral outer capsid protein VP8, which is subject to higher regional and temporal variation [54]. The functions of the proteins encoded by the other nine segments have yet to be investigated and database homology searches do not suggest a function. Genetic analyses based on dN/dS and neutrality tests may provide some insight into their function or constraints on their variability or selection. Segments seg7 and seg10, with higher dN/dS, might encode proteins subject to periodic positive selection, such as capsid proteins.

The analysis of each individual segment in a multi-segmented genome can provide information on the evolutionary history of that specific segment. Genotypes of NC_ 2019#3 and FL_2019#2 grouped distinctly from other US Atlantic coast strains in the concatenated genome analysis primarily due to a relatively high number of nucleotide differences in seg3, seg5, seg8, and seg10, but the uniqueness of these genetic differences was not reflected in the analysis of other segments. Reassortment or recombination between divergent sequence variants can generate new viral genotypes and accelerate the evolution of segmented RNA viruses [97,98]. Due to reassortment processes in viruses with segmented genomes, individual genomic segments can have different evolutionary histories and therefore show different tree topologies [99]. There were several CsRV1 genotypes that showed topological variation among the segment trees, including the genotypes from the GoM and CAR. These variants had polytomous topology on most segment trees and formed well-supported nodes with either the USAC (seg8) or the SAmer (seg1, seg2, and seg5) variants. However, there were too few full CsRV1 genome sequences available for locations in the CAR to draw firm conclusions about reassortment and recombination in this study. Reassortment, recombination, and the evolutionary origins of CsRV1 may become more evident as more CsRV1 genotypes are sequenced in the future.

## 5. Conclusions

This study revealed patterns of genetic structure and genetic diversity of CsRV1 at a trans-hemispheric scale, through the lens of how evolutionary dynamics of CsRV1 may be influenced by interactions between the virus, the host life history, and latitude. Overall, this study sheds light on the factors controlling the spread and connectivity of viral pathogens in marine systems, examining the effect of geographic distance, possible oceanographic barriers and ocean currents, host migration and life history, and potential human-mediated movement of marine animals. Implicit in our study is the idea that for animals with complex life histories, there may not be perfect congruence between pathogen population genetic structure and that of its host. To fully address this question, a corresponding high-resolution study of blue crab genotypes in the same geographic range is being completed. The results show that analyzing virus genomes can inform research on host population connectivity and movement in the ocean with, potentially, higher resolution due to the higher mutation rate of the virus. However, attention must be paid to human-mediated processes, such as the anthropogenetic movement of viral genotypes via interstate transport of infected crabs, to accurately interpret findings. Our results highlight possible risks in moving live marine animals between populations and suggest that fishery resource management plans should include pathogen monitoring and biosecurity elements to limit the unintentional transport of pathogens.

## Figures and Tables

**Table 1:** CsRV1 collected from different geographic locations. CsRV1 samples were collected from locations along the US Atlantic coast, Gulf of Mexico, Caribbean Sea, and Atlantic coast of South America during 2015–2019. Genome IDs for both viral seg9 and concatenated genome included in phylogenetic analyses were also shown.

Location	Latitude	Longitude	Collection Year	Seg9 Genome ID	Concatenated Genome ID
**US Atlantic coast**					
Westport River, MA	41.5118° N	71.0929° W	2018	MA 2018 #1–3	MA 2018 #1;2
Long Island, NY	40.9395° N	72.2304° W	2011–2021	NY 2018 #1–6 NY 2020 #1–4	NY 2018 #4 NY 2020 #1
Delaware Bay, DE	38.9108° N	75.5277° W	2017	DE 2017 #1–6	DE 2017 #2;4
Rhode River, MD	38.8648° N	76.5146° W	2006–2020	MD 2020 #1–2	MD 2020 #1
Chesapeake Bay, VA Albemarle Sound, NC Jacksonville, FL Gulf of Mexico Port Aransas, TX Dulac, LA Florida Keys, FLK Caribbean Sea Dominican Republic, DR Puerto Rico, PR Trinidad and Tobago, T&T South America Rio Grande do Sul, BR Uruguay, UY	38.0214° N 33.8772° N 30.3322° N 27.8006° N 29.3888° N 24.8234° N 18.4511° N 18.4508° N 10.4633° N 30.0346° S 34.6285° S	76.3524° W 76.1248° W 81.6557° W 97.3964° W 90.7140° W 80.8122° W 69.2133° W 65.9801° W 61.4836° W 51.2177° W 54.2921° W	2018 2019 2018 2017 2019 2018 2018 2015 2017 2015 2019	VA 2018 #1–2 NC 2019 #1–3 FL 2018 #1–5 TX 2017 #1–4 LA 2019 #1–11 FLK 2018 #1 DR 2018 #1 PR 2015 #1–2 T&T 2017 #1 BR 2015 #1–9 UY 2019 #1–6	VA 2018 #1;2 NC 2019 #3 FL 2018 #2 TX 2017 #2 LA 2019 #1 FLK 2018 #1 NA NA T&T 2017 #1 BR 2015 #3;4;7;8 UY 2019 #1;5;6

**Table 2:** Population genetic diversity indices based on seg9 (843 nt) of 96 CsRV1 within regions of USAC, GoM, CAR, and SAmer. Nt ID (%): percent identity of nucleotides; AA ID (%): percent identity of amino acids; H: haplotype; Hd: Haplotype identity; π: nucleotide diversity; ω (dN/dS): ratio of non-synonymous (dN) to synonymous (dS) substitution; θ: Theta (per site) from Eta (average mutation rates).

Dataset (Individual Number)	Nt ID% (Min–Max)	AA ID% (Min–Max)	H	Hd	π	ω (dN/dS)	θ
USAC + GoM + CAR + SAmer (96)	95.73–100	94.31–100	67	0.98596	0.0140	0.17	0.029
USAC (61)	98.58–100	97.86–100	44	0.98251	0.0078	0.10	0.014
GoM (16)	96.44–100	94.66–100	12	0.94167	0.0135	0.29	0.019
CAR (4)	96.09–100	96.44–100	4	1.00000	0.0172	0.15	0.016
SAmer (15)	99.29–100	98.93–100	8	0.83810	0.0037	0.14	0.004

**Table 3:** Genetic diversity indices and selection pressure based on segment nucleotide sequences in concatenated genomes of CsRV1. % Nt ID: percent identity of nucleotides; % AA ID: percent identity of amino acids; π: nucleotide diversity; ω (dN/dS): ratio of non-synonymous (dN) to synonymous (dS) substitution. S: number of polymorphic sites; k: average number of nucleotide differences; θ: Theta (per site) from Eta (average mutation rates).

Segment	Length (nt)	N of Sequences	% Nt ID (Min–Max)	% AA ID (Min–Max)	π	ω (dN/dS)	s	k	θ
Seg1 Seg2 Seg3 Seg4 Seg5 Seg6 Seg7 Seg8 Seg9 Seg10 Seg11 Seg12	4239 2337 2220 1959 1824 1620 1260 897 972 1032 612 837	23 23 23 23 23 23 23 23 23 23 23 23	97.3–99.7 97.1–99.9 97.6–99.9 96.8–99.9 96.2–100 97.3–99.9 96.3–100 96.4–100 96.1–100 95.2–100 97.7–100 97.6–100	97.5–99.9 97.1–99.0 97.4–100 95.7–99.9 95.2–100 95.9–100 94.3–100 93.6–100 93.8–100 90.7–100 97.0–100 96.4–100	0.014 0.012 0.012 0.015 0.016 0.017 0.021 0.018 0.018 0.021 0.010 0.011	0.11 0.12 0.08 0.19 0.15 0.21 0.30 0.40 0.18 0.37 0.08 0.14	352 207 163 172 170 140 126 74 87 125 42 63	60.23 28.95 26.99 30.15 28.89 28.15 26.81 16.24 17.47 22.29 5.68 9.04	0.023 0.025 0.020 0.025 0.027 0.025 0.031 0.025 0.026 0.035 0.019 0.021

**Table 4:** Pairwise mismatch distributions (SSD and HRI) and neutrality tests (Fu and Li’s D, Fu and Li’s F, Fu’s Fs, and Tajima’s D) based on segment ORF nucleotide sequences. Lengths of each segment were the same as in Table 3.

Segment	N of Sequences	SSD ^a^	HRI ^a^	Tajima’s D	Fu and Li’s D	Fu and Li’s F	Fu’s Fs ^d^
Seg1 Seg2 Seg3 Seg4 Seg5 Seg6 Seg7 Seg8 Seg9 Seg10 Seg11 Seg12	23 23 23 23 23 23 23 23 23 23 23 23	0.0056 0.0051 0.0042 0.0060 0.0181 0.0039 0.0055 0.0133 0.0078 0.0079 0.0050 0.0049	0.0061 0.0091 0.0070 0.0122 0.0080 0.0098 0.0094 0.0102 0.0061 0.0095 0.0194 0.0071	−1.58 −2.03 ^b^ −1.61 −1.54 −1.60 −1.18 −1.18 −1.07 −1.22 −1.57 −1.94 −1.89	−2.60 ^b^ −3.14 ^c^ −2.63 ^b^ −2.54 ^b^ −2.28 −1.87 −1.78 −1.30 −2.04 −1.95 −3.07 ^c^ −2.54 ^b^	−2.68 ^b^ −3.28 ^c^ −2.71 ^b^ −2.61 ^b^ −2.43 −1.94 −1.87 −1.44 −2.09 −2.15 −3.19 ^c^ −2.74 ^b^	−3.73 −7.08 −7.50 −6.84 −4.81 −7.24 −5.19 −8.36 −5.83 −6.15 −5.70 −6.95

^a^: not significant *p*-values. ^b^: *p* < 0.05; ^c^: *p* < 0.02; ^d^: none of the statistics gave significant *p*-value.

**Table 5:** Population genetic differentiation analysis (F*ST*) based on seg9 (843 nt) (middle) between regions and between subregions within the US. Asterisk * indicates *p* < 0.05 for all F*ST* estimates. Numbers inside brackets indicate the number of virus strains from that location.

Segment	Subpopulation 1	Subpopulation 2	F*ST* *
Seg9	USAC (61)	SAmer (15)	0.69468
(All regions)	USAC (61)	GoM (16)	0.16164
	USAC (61)	CAR (4)	0.66668
	GoM (16)	CAR (4)	0.51389
	GoM (16)	SAmer (15)	0.63118
	CAR (4)	SAmer (15)	0.62649
Seg9 (Within US)	N. Atl (25) N. Atl (25) N. Atl(25) M. Atl (31) M. Atl (31) S. Atl (5)	M. Atl (31) S. Atl (5) GoM (16) S. Atl (5) GoM (16) GoM (16)	0.22894 0.57072 0.25073 0.47729 0.14819 0.35522

**Table 6:** Analysis of molecular variance (AMOVA) for CsRV1 among regions, subregions within the US, and locations within the SAmer, based on nucleotide sequences of seg1 (4239 nt), seg8 (897 nt), and seg9 (843 nt). Among all regions: US (USAC and GoM), CAR, and SAmer. Among subregions within the US: N. Atl, M. Atl, S. Atl, and GoM. Among locations within the SAmer: BR and UY.

Segment	Source of Variance	d.f.	Sum of Squares	Variance Component	Percentage of Variation	AMOVA Statistics	*p*-Value
Seg1 (All regions)	Among populations Within populations Total	2 20 22	266.880 395.686 662.565	20.58327 Va 19.78429 Vb 40.36756	50.99 49.01	0.50990	0.00
Seg8	Among populations	2	84.519	6.80089 Va	59.10	0.59099	0.00
(All regions)	Within populations	20	94.133	4.70667 Vb	40.90		
	Total	20	178.652	11.50756			
Seg9	Among populations	2	212.912	6.47360 Va	63.29	0.63290	0.00
(All regions)	Within populations	93	349.203	3.75487 Vb	36.71		
	Total	95	562.115	10.22847			
Seg9	Among populations	3	71.109	1.17949 Va	26.76	0.26764	0.00
(Within the US)	Within populations	72	232.378	3.22747 Vb	73.24		
	Total	75	303.487	4.40696			
Seg9	Among populations	1	3.544	0.29582 Va	17.30	0.17296	0.05
(Within the SAmer)	Within populations	13	18.389	1.41453 Vb	82.70		
	Total	14	21.933	1.71035			

## Data Availability

The sequences generated in this study are deposited in NCBI GenBank, with accession numbers OP067244-OP067635.

## Supplementary Materials

The following supporting information can be downloaded at: https://www.mdpi.com/article/10.3390/v15020563/s1, Figure S1: Pairwise comparisons of CsRV1 concatenated whole genome based on nucleotide sequences (top) and amino acid sequences (bottom); Figure S2: Maximum Likelihood phylogenetic tree based on amino acid sequences of the concatenated genomes(A) and segment 9 (B) of CsRV1 isolates from different geographic locations; Figure S3: Maximum likelihood phylogenetic tree based on the nucleotide sequences of each segment of 23 strains of CsRV1 collected from different geographic locations. Table S1: Primers used in this study; Table S2: *F*_ST_ estimates of CsRV1 between locations; Table S3 Sequence length and best models based on BIC for constructing Maximum Likelihood phylogenetic tree for each genomic segment of CsRV1.
