# Supplementary material for: Cross-Hemispheric Genetic Diversity and Spatial Genetic Structure of Callinectes sapidus Reovirus 1 (CsRV1)
Source: Viruses. 2023 Feb 18;15(2):563. doi: 10.3390/v15020563 (PMC9962310; doi:10.3390/v15020563)
Supplement: Supplementary file 1 [file viruses-15-00563-s001.zip › viruses-2130485-Figure and Tables.pdf]

Supplementary Figure S1. Pairwise comparisons of CsRV1 concatenated whole genome based on nucleotide sequences (top) and amino acid sequences (bottom).

		1	2	3	4	5	6	7	8	9	10	11	12	13	14	15	16	17	18	19	20	21	22	23
MD_2008__X45	1		99.08	99.32	99.44	99.26	99.26	99.20	99.30	99.11	99.29	98.64	98.53	98.66	98.46	97.77	97.87	98.56	98.50	98.42	98.44	98.44	98.36	98.48
MD_2020__1	2	182		99.15	98.98	98.89	98.95	99.10	99.21	99.09	99.18	98.84	98.70	98.69	98.22	97.54	97.59	98.28	98.23	98.17	98.18	98.17	98.08	98.21
MA_2018__1	3	134	169		99.31	99.00	99.24	99.34	99.47	99.32	99.44	98.68	98.51	98.63	98.30	97.69	97.73	98.41	98.38	98.31	98.35	98.36	98.24	98.35
MA_2018__2	4	110	202	136		99.15	99.29	99.16	99.28	99.10	99.25	98.58	98.50	98.59	98.38	97.72	97.83	98.47	98.41	98.35	98.35	98.36	98.26	98.39
NY_2020__1	5	146	219	198	168		99.46	98.90	98.95	98.99	99.00	98.60	98.49	98.51	98.41	97.73	97.81	98.52	98.47	98.41	98.40	98.40	98.32	98.45
NY_2018__4	6	146	208	150	141	107		99.05	99.12	99.14	99.14	98.65	98.56	98.58	98.46	97.79	97.89	98.56	98.51	98.45	98.44	98.45	98.35	98.48
VA_2018__1	7	159	179	131	167	217	189		99.27	99.21	99.40	98.55	98.37	98.48	98.19	97.53	97.60	98.28	98.23	98.16	98.17	98.17	98.08	98.18
VA_2018__2	8	139	156	105	143	208	174	144		99.38	99.37	98.73	98.62	98.68	98.29	97.65	97.71	98.40	98.35	98.29	98.30	98.29	98.20	98.31
DE_2017__4	9	177	180	134	178	201	170	157	123		99.38	98.63	98.51	98.59	98.23	97.63	97.64	98.34	98.29	98.25	98.26	98.24	98.13	98.25
DE_2017__2	10	140	162	111	148	198	171	119	125	123		98.72	98.50	98.64	98.30	97.65	97.70	98.38	98.33	98.27	98.30	98.28	98.17	98.28
NC_2019__3	11	269	229	261	281	277	268	287	251	272	254		98.89	98.64	98.05	97.35	97.41	98.08	98.00	97.96	97.96	97.97	97.86	97.96
FL_2018__2	12	292	258	295	298	300	285	323	274	296	298	219		98.59	97.99	97.34	97.32	97.99	97.94	97.87	97.87	97.87	97.77	97.90
LA_2019__1	13	265	260	271	279	296	281	302	261	280	269	269	279		98.23	97.52	97.56	98.29	98.23	98.19	98.21	98.17	98.09	98.20
TX_2017__2	14	305	353	336	321	314	305	358	339	351	337	387	398	350		97.79	97.78	98.52	98.49	98.42	98.44	98.41	98.33	98.45
FLK_2018__1	15	442	488	458	451	450	438	489	466	470	466	524	527	491	438		97.41	97.76	97.74	97.75	97.70	97.69	97.68	97.77
T&T_2017__1	16	422	477	450	429	433	418	475	453	467	456	514	530	484	439	514		97.91	97.88	97.87	97.82	97.82	97.76	97.89
BR_2015__3	17	285	341	314	303	293	286	341	317	328	320	381	399	339	293	443	414		99.67	99.49	99.47	99.36	99.33	99.44
BR_2015__4	18	297	351	320	315	303	296	350	327	338	330	396	409	350	300	447	419	65		99.59	99.55	99.41	99.40	99.52
BR_2015__7	19	312	363	334	326	315	307	365	338	347	343	404	422	359	312	445	422	101	82		99.57	99.44	99.43	99.55
BR_2015__8	20	310	361	327	326	317	309	363	336	345	336	405	421	354	310	456	431	105	90	86		99.37	99.34	99.45
UY_2019__1	21	309	362	324	325	317	308	362	338	348	340	402	422	363	314	457	432	126	117	111	124		99.57	99.62
UY_2019__5	22	325	380	348	345	332	326	380	357	370	362	423	442	379	330	460	444	132	119	113	130	86		99.70
UY_2019__6	23	302	355	327	319	307	301	360	335	346	340	404	416	356	308	442	418	111	96	90	109	76	60	

		1	2	3	4	5	6	7	8	9	10	11	12	13	14	15	16	17	18	19	20	21	22	23
MD_2008_#X45	1		99.06	99.44	99.56	99.38	99.32	99.43	99.35	99.11	99.41	98.67	98.37	98.26	97.75	97.35	97.57	98.05	97.96	97.87	97.88	98.05	97.90	97.97
MD_2020_#1	2	62		99.15	98.96	98.87	98.87	99.18	99.18	99.02	99.21	99.06	98.75	98.38	97.57	97.07	97.28	97.76	97.67	97.64	97.66	97.76	97.61	97.72
MA_2018_#1	3	37	56		99.38	99.08	99.18	99.53	99.56	99.30	99.55	98.70	98.40	98.23	97.67	97.32	97.44	97.99	97.93	97.84	97.88	97.99	97.84	97.91
MA_2018_#2	4	29	69	41		99.29	99.35	99.38	99.27	99.02	99.40	98.61	98.34	98.14	97.67	97.31	97.54	97.99	97.87	97.81	97.79	97.96	97.81	97.91
NY_2020_#1	5	41	75	61	47		99.65	99.06	98.97	98.88	99.08	98.62	98.37	98.05	97.76	97.38	97.54	98.05	97.96	97.90	97.88	98.02	97.87	97.97
NY_2018_#4	6	45	75	54	43	23		99.08	99.03	98.94	99.08	98.61	98.40	98.08	97.79	97.44	97.61	98.11	98.02	97.96	97.94	98.08	97.93	98.03
VA_2018_#1	7	38	54	31	41	62	61		99.43	99.27	99.59	98.68	98.38	98.17	97.61	97.26	97.40	97.91	97.84	97.75	97.76	97.90	97.75	97.82
VA_2018_#2	8	43	54	29	48	68	64	38		99.47	99.41	98.73	98.52	98.35	97.61	97.26	97.41	97.94	97.85	97.76	97.81	97.91	97.76	97.84
DE_2017_#4	9	59	65	46	65	74	70	48	35		99.38	98.53	98.35	98.16	97.44	97.11	97.22	97.78	97.69	97.63	97.64	97.72	97.57	97.67
DE_2017_#2	10	39	52	30	40	61	61	27	39	41		98.75	98.38	98.29	97.63	97.25	97.41	97.94	97.85	97.76	97.81	97.91	97.76	97.84
NC_2019_#3	11	88	62	86	92	91	92	87	84	97	83		99.12	98.50	97.57	97.04	97.16	97.63	97.52	97.46	97.51	97.67	97.49	97.57
FL_2018_#2	12	108	83	106	110	108	106	107	98	109	107	58		98.37	97.37	96.89	97.01	97.35	97.31	97.16	97.20	97.31	97.19	97.29
LA_2019_#1	13	115	107	117	123	129	127	121	109	122	113	99	108		97.35	96.79	96.87	97.44	97.37	97.34	97.35	97.49	97.32	97.41
TX_2017_#2	14	149	161	154	154	148	146	158	158	169	157	161	174	175		97.13	97.16	97.79	97.72	97.63	97.69	97.78	97.57	97.70
FLK_2018_#1	15	175	194	177	178	173	169	181	181	191	182	196	206	212	190		97.11	97.34	97.23	97.20	97.20	97.37	97.26	97.35
T&T_2017_#1	16	161	180	169	163	163	158	172	171	184	171	188	198	207	188	191		97.51	97.41	97.37	97.37	97.48	97.29	97.43
BR_2015_#3	17	129	148	133	133	129	125	138	136	147	136	157	175	169	146	176	165		99.59	99.36	99.41	99.40	99.24	99.35
BR_2015_#4	18	135	154	137	141	135	131	143	142	153	142	164	178	174	151	183	171	27		99.56	99.49	99.44	99.32	99.43
BR_2015_#7	19	141	156	143	145	139	135	149	148	157	148	168	188	176	157	185	174	42	29		99.53	99.52	99.40	99.50
BR_2015_#8	20	140	155	140	146	140	136	148	145	156	145	165	185	175	153	185	174	39	34	31		99.40	99.27	99.38
UY_2019_#1	21	129	148	133	135	131	127	139	138	151	138	154	178	166	147	174	167	40	37	32	40		99.58	99.65
UY_2019_#5	22	139	158	143	145	141	137	149	148	161	148	166	186	177	161	181	179	50	45	40	48	28		99.65
UY_2019_#6	23	134	151	138	138	134	130	144	143	154	143	161	179	171	152	175	170	43	38	33	41	23	23	

Supplementary Figure S2. Maximum Likelihood phylogenetic tree based on amino acid sequences of the concatenated genomes(A) and segment 9 (B) of CsRV1 isolates from different geographic locations. Bootstrap > 50% was shown on the tree. SsRV WX_2012 was used as the outgroup.

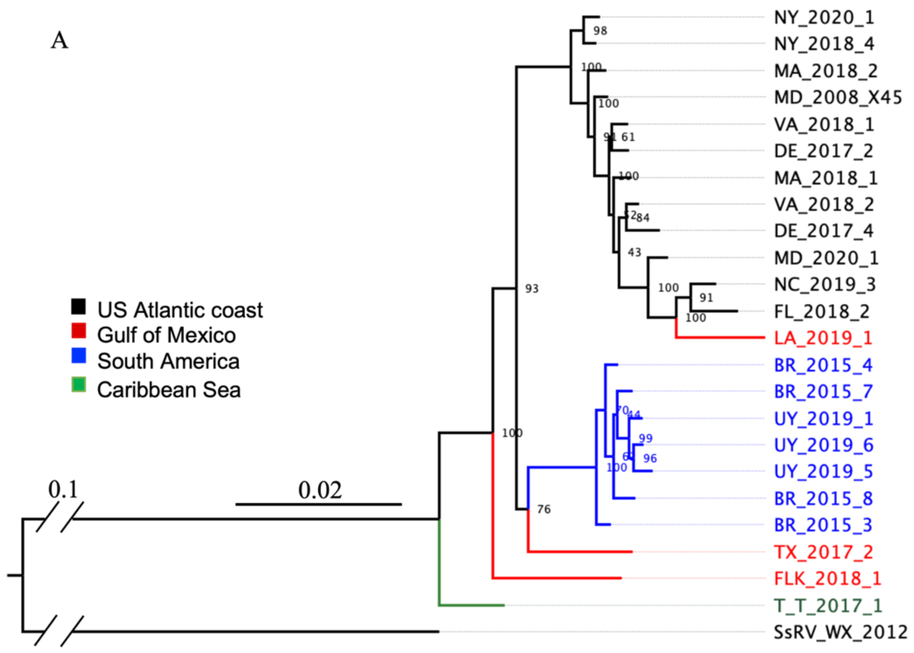
A
US Atlantic coast
Gulf of Mexico
South America
Caribbean Sea
0.1
0.02
NY_2020_1
NY_2018_4
MA_2018_2
MD_2008_X45
VA_2018_1
DE_2017_2
MA_2018_1
VA_2018_2
DE_2017_4
MD_2020_1
NC_2019_3
FL_2018_2
LA_2019_1
BR_2015_4
BR_2015_7
UY_2019_1
UY_2019_6
UY_2019_5
BR_2015_8
BR_2015_3
TX_2017_2
FLK_2018_1
T_T_2017_1
SsRV_WX_2012

B

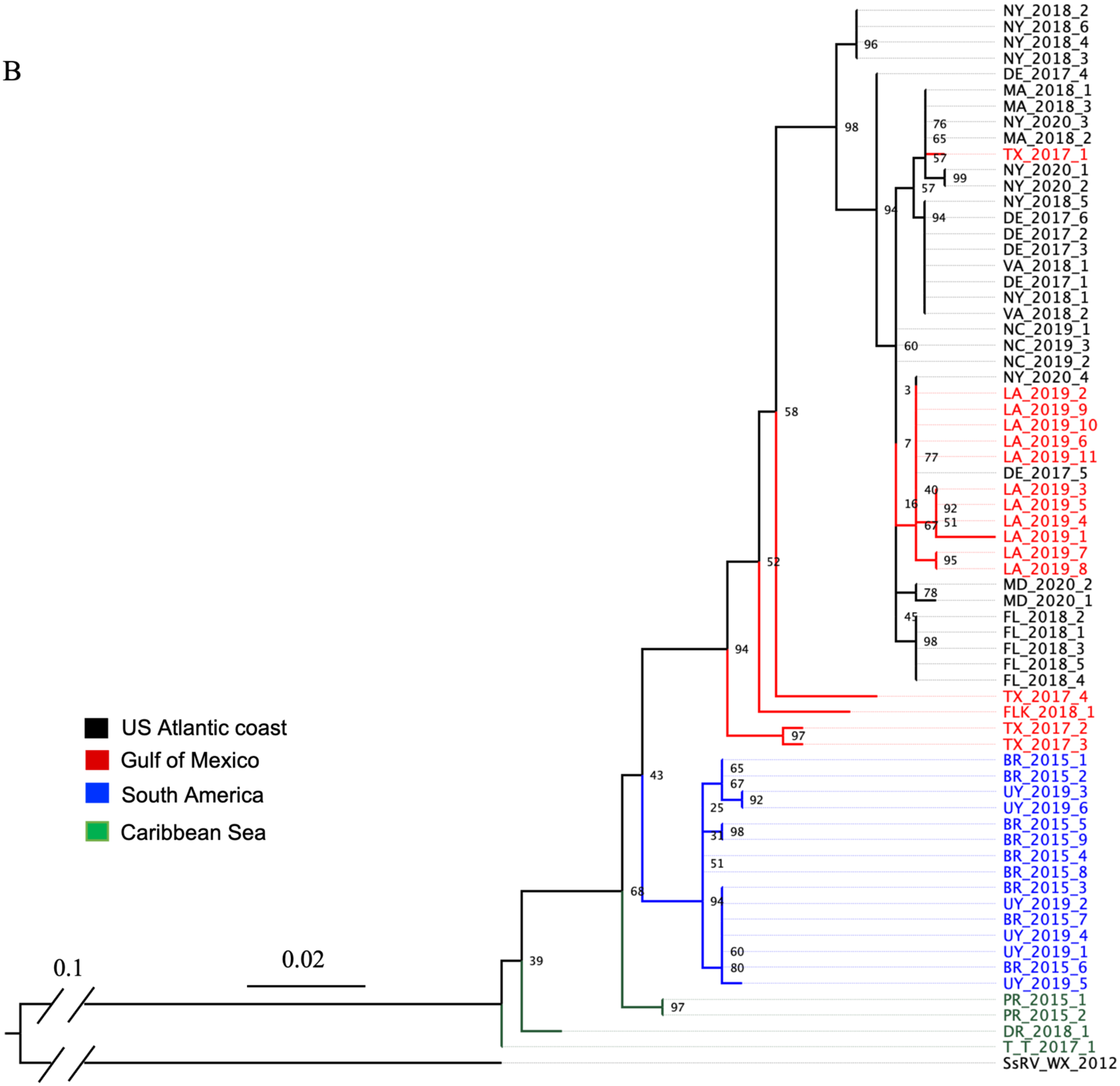

Supplementary Figure S3. Maximum likelihood phylogenetic tree based on the nucleotide sequences of each segment of 23 strains of CsRV1 collected from different geographic locations. Best Models based on BIC for each tree were shown in Supplementary Table 3.

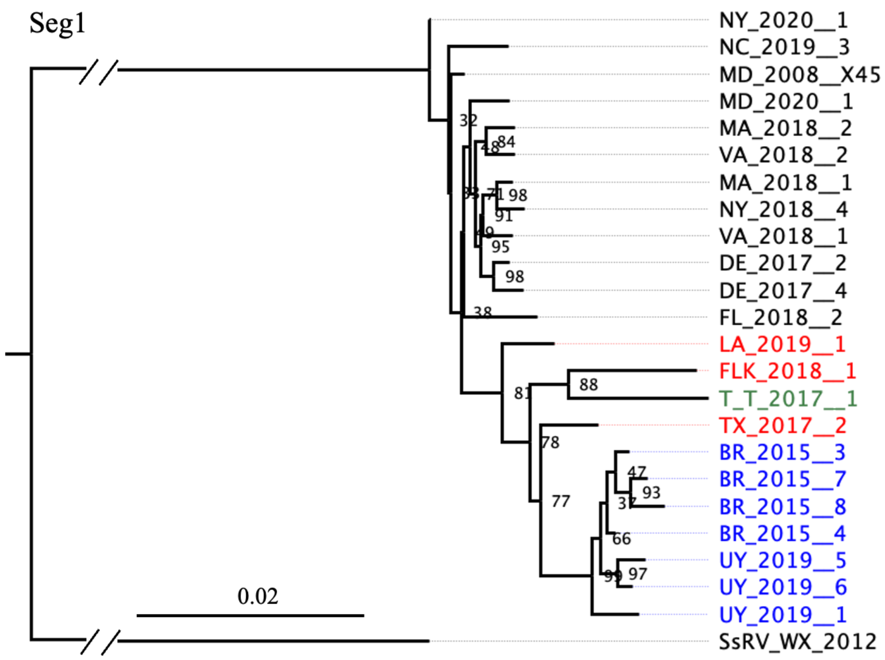
Seg1
NY_2020__1
NC_2019__3
MD_2008__X45
MD_2020__1
MA_2018__2
VA_2018__2
MA_2018__1
NY_2018__4
VA_2018__1
DE_2017__2
DE_2017__4
FL_2018__2
LA_2019__1
FLK_2018__1
T_T_2017__1
TX_2017__2
BR_2015__3
BR_2015__7
BR_2015__8
BR_2015__4
UY_2019__5
UY_2019__6
UY_2019__1
SsRV_WX_2012
0.02
32
84
98
91
95
98
38
88
81
78
77
47
93
66
97

Seg 2

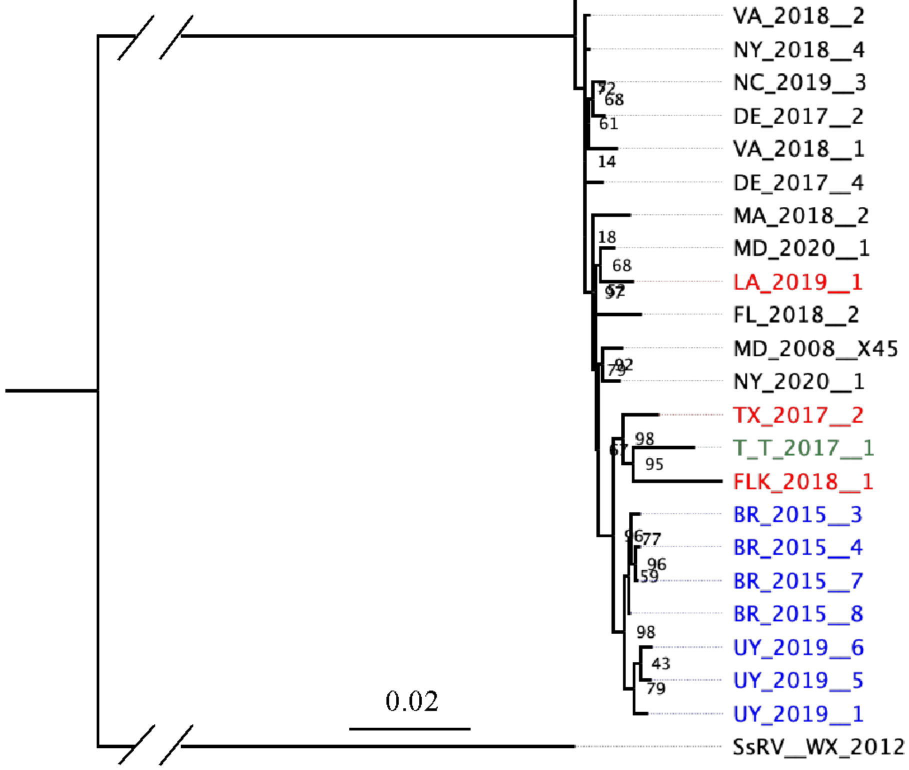
MA_2018__1
VA_2018__2
NY_2018__4
NC_2019__3
DE_2017__2
VA_2018__1
DE_2017__4
MA_2018__2
MD_2020__1
LA_2019__1
FL_2018__2
MD_2008__X45
NY_2020__1
TX_2017__2
T_T_2017__1
FLK_2018__1
BR_2015__3
BR_2015__4
BR_2015__7
BR_2015__8
UY_2019__6
UY_2019__5
UY_2019__1
SsRV__WX_2012
0.02

Seg3

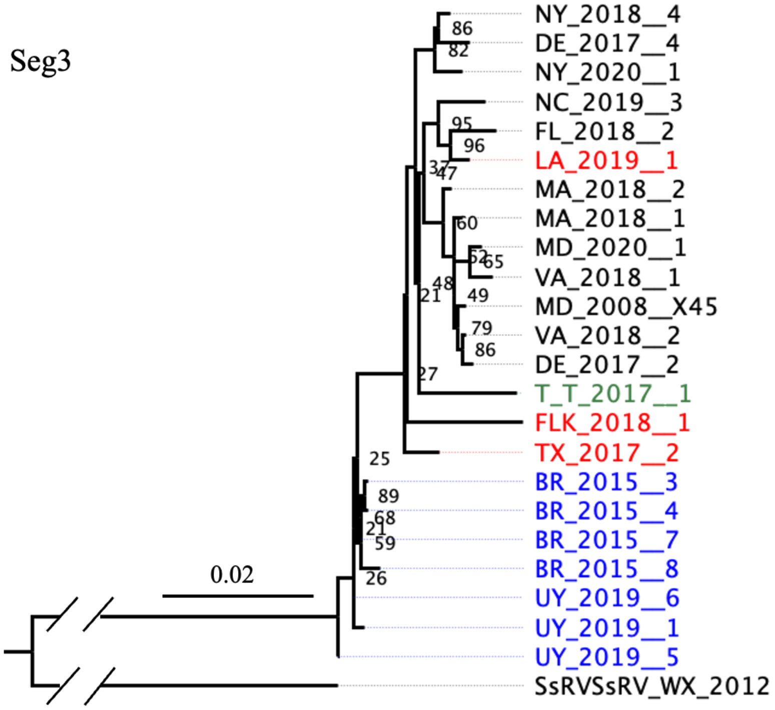
NY_2018__4
DE_2017__4
NY_2020__1
NC_2019__3
FL_2018__2
LA_2019__1
MA_2018__2
MA_2018__1
MD_2020__1
VA_2018__1
MD_2008__X45
VA_2018__2
DE_2017__2
T_T_2017__1
FLK_2018__1
TX_2017__2
BR_2015__3
BR_2015__4
BR_2015__7
BR_2015__8
UY_2019__6
UY_2019__1
UY_2019__5
SsRVSsRV_WX_2012
0.02

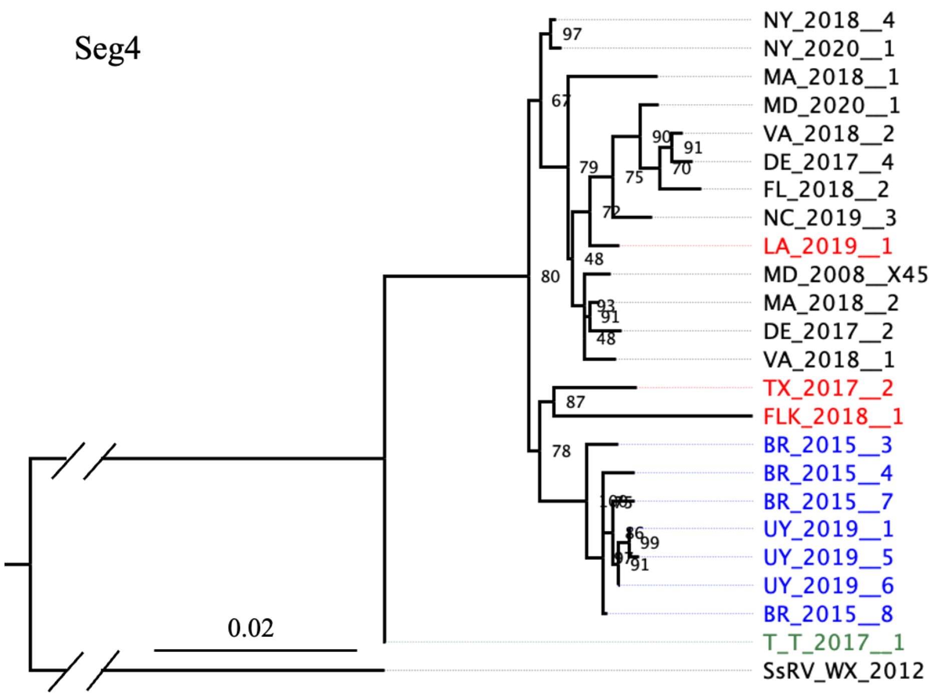
Seg4
NY_2018__4
NY_2020__1
MA_2018__1
MD_2020__1
VA_2018__2
DE_2017__4
FL_2018__2
NC_2019__3
LA_2019__1
MD_2008__X45
MA_2018__2
DE_2017__2
VA_2018__1
TX_2017__2
FLK_2018__1
BR_2015__3
BR_2015__4
BR_2015__7
UY_2019__1
UY_2019__5
UY_2019__6
BR_2015__8
T_T_2017__1
SsRV_WX_2012
0.02

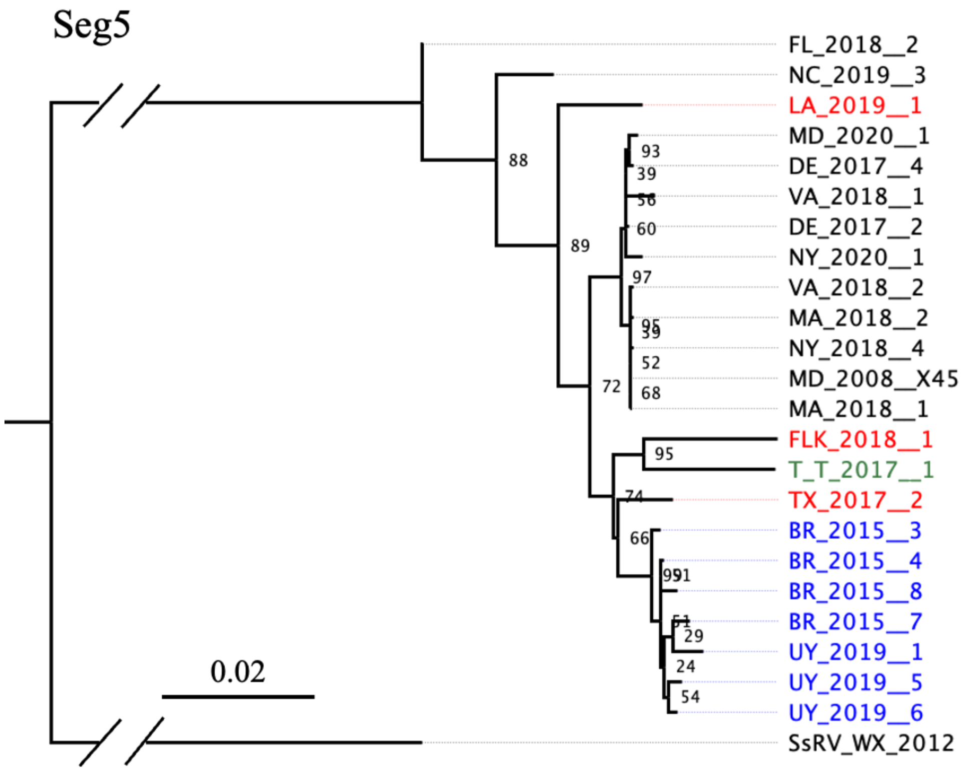
Seg5
FL_2018__2
NC_2019__3
LA_2019__1
MD_2020__1
DE_2017__4
VA_2018__1
DE_2017__2
NY_2020__1
VA_2018__2
MA_2018__2
NY_2018__4
MD_2008__X45
MA_2018__1
FLK_2018__1
T_T_2017__1
TX_2017__2
BR_2015__3
BR_2015__4
BR_2015__8
BR_2015__7
UY_2019__1
UY_2019__5
UY_2019__6
SsRV_WX_2012
0.02

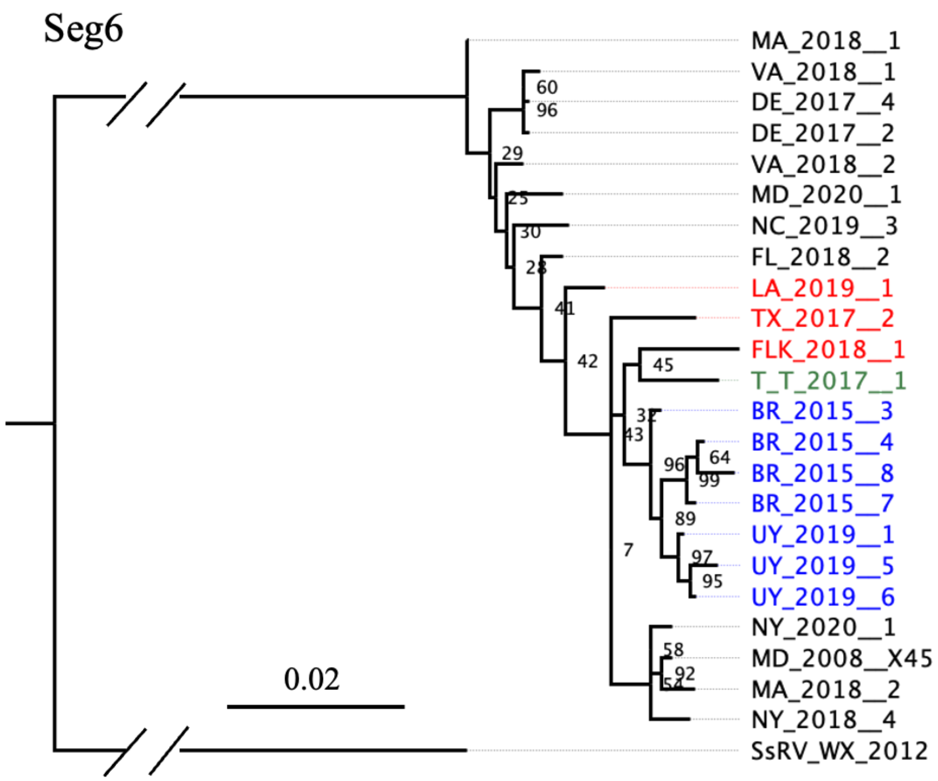
Seg6
MA_2018__1
VA_2018__1
DE_2017__4
DE_2017__2
VA_2018__2
MD_2020__1
NC_2019__3
FL_2018__2
LA_2019__1
TX_2017__2
FLK_2018__1
T_T_2017__1
BR_2015__3
BR_2015__4
BR_2015__8
BR_2015__7
UY_2019__1
UY_2019__5
UY_2019__6
NY_2020__1
MD_2008__X45
MA_2018__2
NY_2018__4
SsRV_WX_2012
0.02

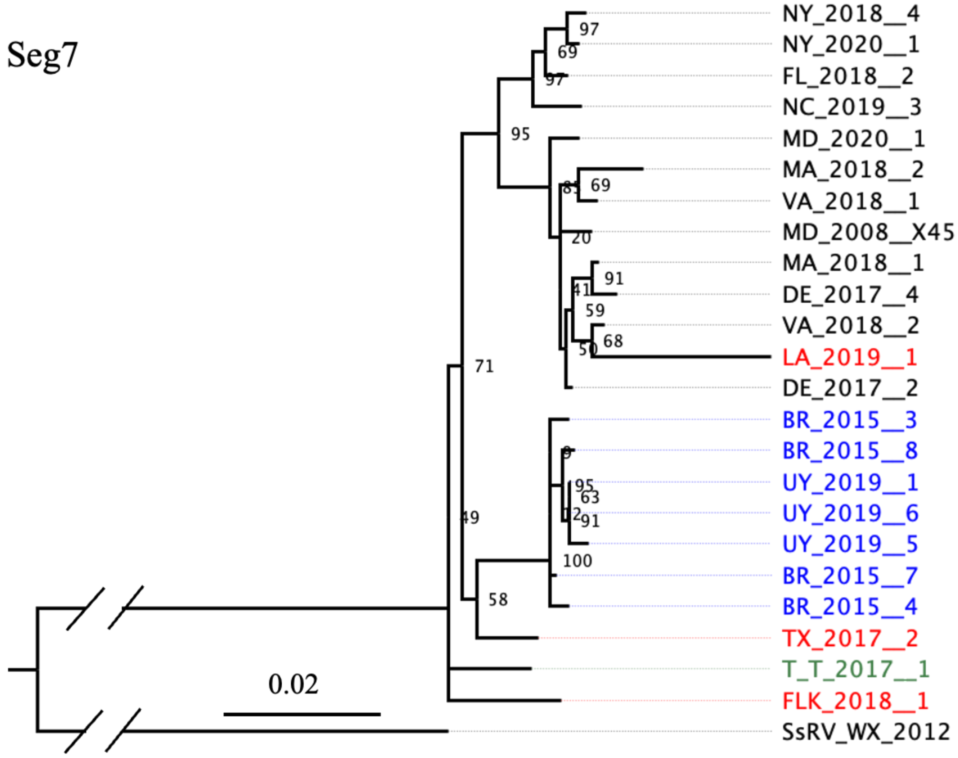
Seg7
NY_2018__4
NY_2020__1
FL_2018__2
NC_2019__3
MD_2020__1
MA_2018__2
VA_2018__1
MD_2008__X45
MA_2018__1
DE_2017__4
VA_2018__2
LA_2019__1
DE_2017__2
BR_2015__3
BR_2015__8
UY_2019__1
UY_2019__6
UY_2019__5
BR_2015__7
BR_2015__4
TX_2017__2
T_T_2017__1
FLK_2018__1
SsRV_WX_2012
0.02

Seg8

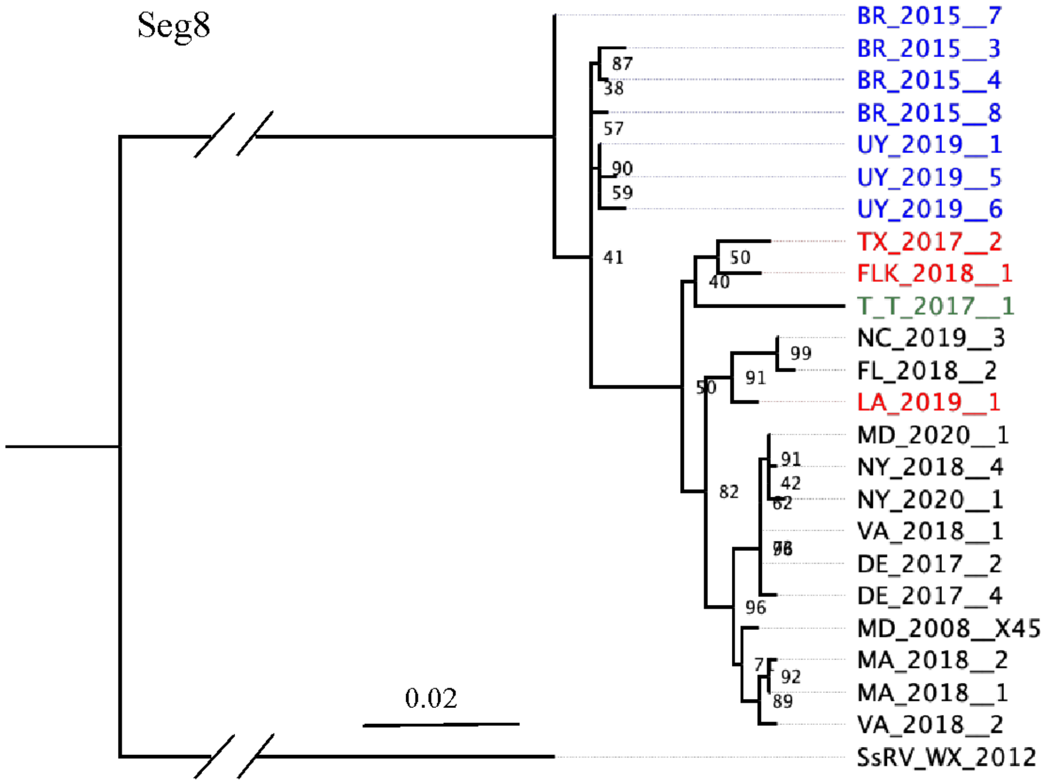
BR_2015__7
BR_2015__3
BR_2015__4
BR_2015__8
UY_2019__1
UY_2019__5
UY_2019__6
TX_2017__2
FLK_2018__1
T_T_2017__1
NC_2019__3
FL_2018__2
LA_2019__1
MD_2020__1
NY_2018__4
NY_2020__1
VA_2018__1
DE_2017__2
DE_2017__4
MD_2008__X45
MA_2018__2
MA_2018__1
VA_2018__2
SsRV_WX_2012
0.02

Seg9

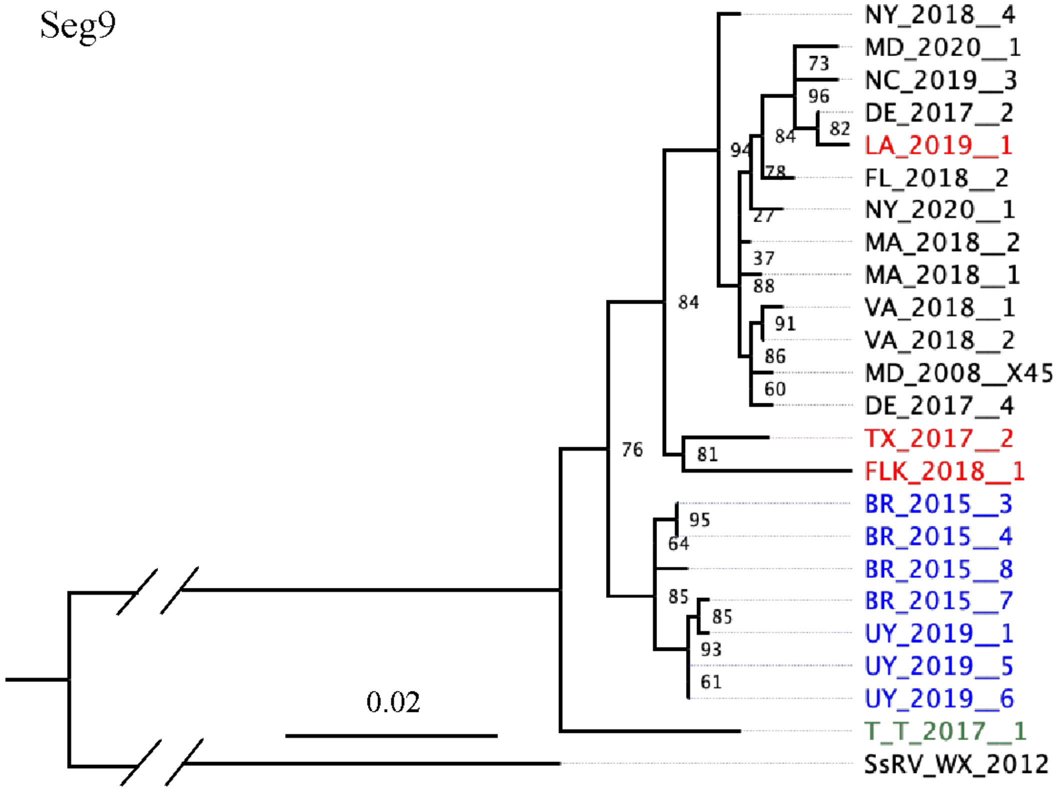
NY_2018__4
MD_2020__1
NC_2019__3
DE_2017__2
LA_2019__1
FL_2018__2
NY_2020__1
MA_2018__2
MA_2018__1
VA_2018__1
VA_2018__2
MD_2008__X45
DE_2017__4
TX_2017__2
FLK_2018__1
BR_2015__3
BR_2015__4
BR_2015__8
BR_2015__7
UY_2019__1
UY_2019__5
UY_2019__6
T_T_2017__1
SsRV_WX_2012
0.02

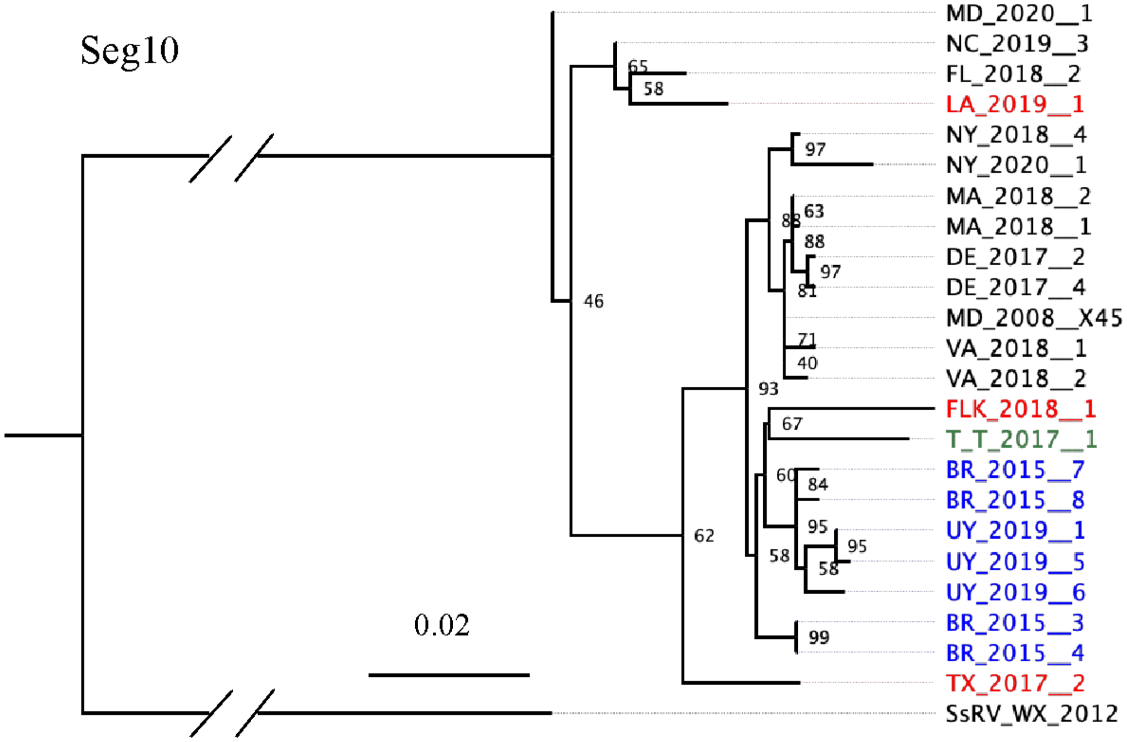
Seg10
MD_2020__1
NC_2019__3
FL_2018__2
LA_2019__1
NY_2018__4
NY_2020__1
MA_2018__2
MA_2018__1
DE_2017__2
DE_2017__4
MD_2008__X45
VA_2018__1
VA_2018__2
FLK_2018__1
T_T_2017__1
BR_2015__7
BR_2015__8
UY_2019__1
UY_2019__5
UY_2019__6
BR_2015__3
BR_2015__4
TX_2017__2
SsRV_WX_2012
0.02

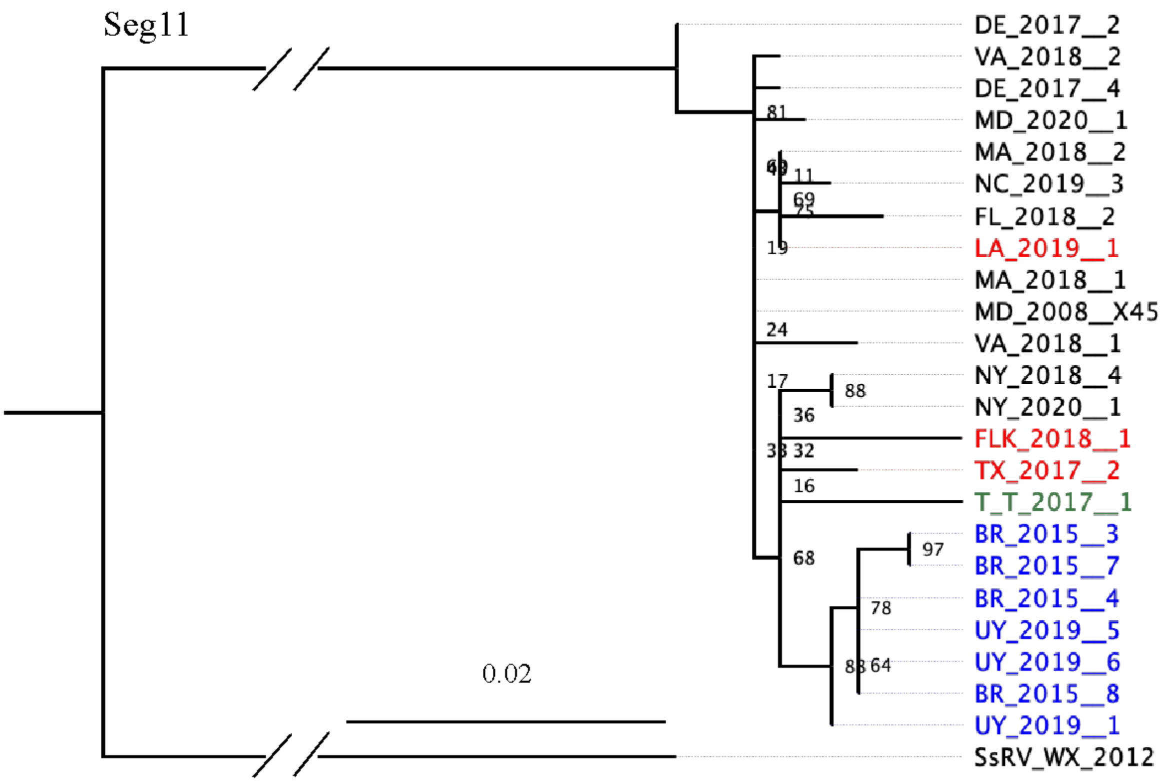

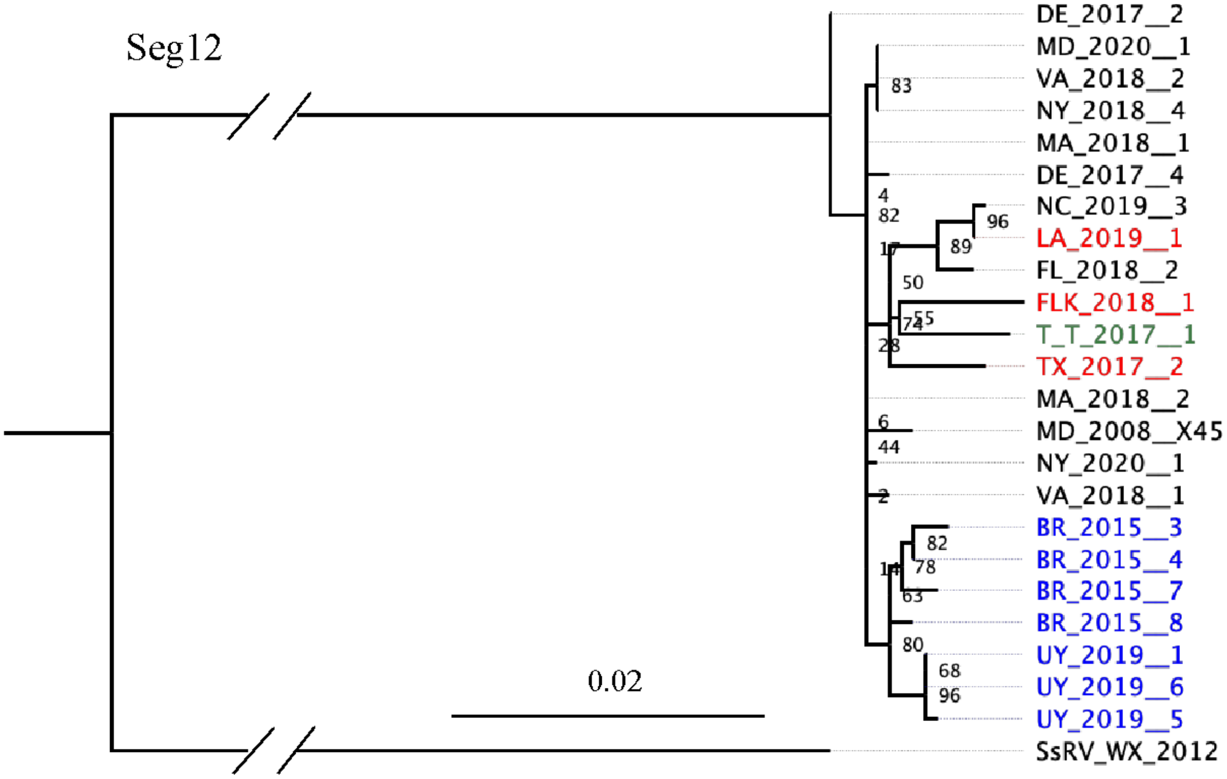

Supplementary Table S1. Primers used in this study.
(See attached excel)

Supplementary Table S2. F_{ST} estimates of CsRV1 between collection years based on seg9. Bold values indicate significance (P < 0.05).

	NY11-13	NY18-21	MD06-09	MD10-12	MD18-20
NY11-13	-				
NY18-21	**0.75**	-			
MD06-09	**0.31**	**0.51**	-		
MD10-12	**0.47**	**0.59**	0.03	-	
MD18-20	**0.40**	**0.54**	-0.08	0.17	-

Supplementary Table S3. F_{ST} estimates of CsRV1 between locations. Bold values indicate significance (P < 0.05).

	MA	NY	VA	DE	MD	NC	LA	FL	TX	CAR	BR	UY
MA	-											
NY	0.06	-										
VA	0.48	0.21	-									
DE	0.15	0.12	0.12	-								
MD	**0.32**	**0.31**	**0.35**	0.17	-							
NC	0.76	**0.47**	0.86	0.31	0.23	-						
LA	**0.67**	**0.52**	**0.73**	**0.43**	**0.34**	0.45	-					
FL	0.88	**0.57**	**0.95**	**0.62**	**0.53**	**0.96**	**0.79**	-				
TX	0.24	**0.42**	0.19	**0.32**	**0.5**	**0.39**	**0.59**	0.52	-			
CAR	0.55	**0.68**	0.52	**0.59**	**0.7**	**0.65**	**0.77**	**0.74**	**0.3**	-		
BR	**0.77**	**0.72**	**0.78**	**0.73**	**0.74**	**0.83**	**0.84**	**0.87**	**0.46**	**0.53**	-	
UY	**0.91**	**0.76**	**0.93**	**0.8**	**0.78**	**0.95**	**0.9**	**0.97**	**0.5**	**0.6**	0.17	-
